# Human-in-the-Loop Artificial Intelligence: A Systematic Review of Concepts, Methods, and Applications

**DOI:** 10.3390/e28040377

**Published:** 2026-03-26

**Authors:** Konstantinos Lazaros, Aristidis G. Vrahatis, Sotiris Kotsiantis

**Affiliations:** 1 Department of Informatics, Ionian University, 49100 Corfu, Greece; 2 Department of Mathematics, University of Patras, 26504 Patras, Greece

**Keywords:** human-in-the-loop, artificial intelligence, machine learning, human–AI collaboration, explainable AI, active learning, reinforcement learning, human oversight

## Abstract

The integration of human judgment into artificial intelligence (AI) systems has emerged as a key research direction, particularly for high-stakes applications where full automation remains insufficient. Human-in-the-Loop (HITL) AI represents a field that combines machine learning capabilities with human oversight, feedback, and decision-making at various stages of the AI pipeline. This survey provides a systematic review of HITL approaches, covering theoretical foundations, technical methods, ethical considerations, and domain-specific applications. We propose a unified taxonomy that categorizes HITL systems based on loop placement, interaction granularity, and temporal characteristics. This review synthesizes findings from healthcare, autonomous systems, cybersecurity, and other high-risk domains where human oversight is essential. We also examine the challenges of scalability, cognitive load, and trust calibration that affect the practical deployment of HITL systems. The final section outlines open research directions and introduces a framework for designing effective human–AI collaborative systems.

## 1. Introduction

Artificial intelligence systems have displayed remarkable performance capabilities over a wide range of tasks, including image recognition and natural language processing. However, despite these developments, practical applications of artificial intelligence systems reveal inherent limitations that cannot be addressed through automation alone. In situations involving sophisticated decision-making processes, such as in healthcare, finance, legal processes, and autonomous systems, understanding contexts, making ethical judgments, and being accountable are requirements that artificial intelligence systems are not yet able to offer. This has led to the development of various Human-in-the-Loop (HITL) methods that involve the integration of human expertise into artificial intelligence systems during various phases of learning processes [1].

The term human-in-the-loop (HITL) extends beyond the simple oversight of automated systems. Modern HITL systems enable a two-way interaction in which human input is incorporated to influence the model’s response. Artificial intelligence systems enable the extension of human capabilities to process large volumes of data to identify patterns that may be difficult for humans to identify on their own. This model has been found to be particularly effective in environments with high error costs and the need for decision-making processes to be explainable [2]. The growing adoption of HITL approaches reflects a broader shift in AI research from pursuing full autonomy toward designing systems that enhance rather than replace human decision-making.

**Figure 1:**
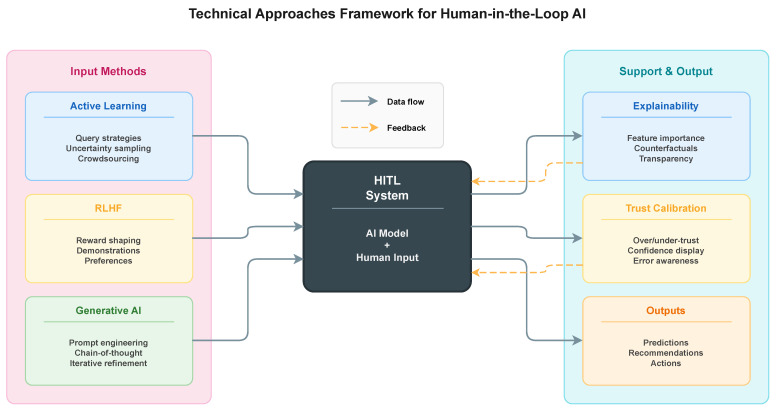
Conceptual framework of Human-in-the-Loop (HITL) AI showing how active learning, RLHF, and generative AI integrate human input into the core model, while explainability and trust calibration support transparent, feedback-driven predictions, recommendations, and actions.

Several factors have contributed to the increased interest in HITL systems over the past decade. The deployment of machine learning models in sensitive domains such as medical diagnosis, criminal justice, and financial lending has raised concerns about algorithmic bias, lack of transparency, and the potential for harmful outcomes when humans are excluded from the decision process [3]. Regulatory frameworks, including the European Union AI Act, now mandate human oversight for high-risk AI applications, creating both legal requirements and practical incentives for HITL design [4]. At the same time, advances in interactive machine learning, active learning, and reinforcement learning from human feedback have provided technical foundations for building effective HITL systems that can learn efficiently from limited human input. Together, these regulatory, ethical, and technical developments motivate the Human-in-the-Loop design space summarized in Figure 1, which outlines the main technical approaches for integrating human input and feedback across the AI pipeline.

The scope of HITL research spans multiple disciplines and application domains. In machine learning, HITL methods address challenges of data annotation, model training, and output validation through structured human involvement [5]. Human–computer interaction research focuses on the ways human–computer interaction can be designed so that human–artificial intelligence collaboration is facilitated, the cognitive load is managed, and user trust is maintained. The domain of AI ethics deals with the issues of responsibility, accountability, and value alignment that arise from human–artificial intelligence co-decision-making. Domain-specific research, for instance, in the domains of healthcare, autonomous vehicles, and cybersecurity, focuses on the ways human-in-the-loop principles can be adapted to the specific domain’s requirements and constraints [6].

Yet, even with the increasing number of publications on human-in-the-loop (HITL) research, the domain is fragmented over a range of disciplines. The results are not well integrated. The existing surveys are found to focus on specific technical approaches (e.g., active learning) or specific application domains (e.g., medical imaging). A unifying framework that connects theory, methods, and applications is absent. This prevents both researchers and practitioners from fully appreciating the range of HITL methodologies and the best approach for their needs [7]. The present survey addresses this gap by providing a systematic review that synthesizes findings across technical and application domains.

This review makes several contributions to the human-in-the-loop (HITL) literature. First, the review proposes a taxonomy for HITL systems, which considers the placement of the human-in-the-loop, the level of interaction, and the temporal aspects of the interaction. This taxonomy provides a framework for comparing the wide range of techniques used for human-in-the-loop systems, as well as the underlying principles for the construction of HITL systems. Second, the review provides a survey of the underlying techniques that enable human–AI collaboration, including active learning, reinforcement learning from human feedback, and explainability. Third, the review considers the ethical and governance implications of human-in-the-loop systems, including fairness, bias, and legal requirements for human oversight [8]. Fourth, we survey applications across multiple high-stakes domains, identifying common patterns and domain-specific adaptations. Fifth, we discuss open challenges and future research directions, including scalability of human oversight, management of conflicting human feedback, and the design of adaptive HITL architectures [9]. To avoid ambiguity in overlapping terminology, Section 2.6 explicitly defines the scope boundaries among HITL AI, human–AI collaboration, IML, XAI, and RLHF.

Summary of contributions:

- We introduce a unified taxonomy for HITL systems organized along three explicit dimensions—loop placement, interaction granularity, and temporal characteristics—and use it consistently to structure the survey.
- We provide two reusable synthesis “anchor” tables: a method-family comparison (Table 1) and a domain-focused comparison (Table 2).
- We consolidate major HITL method families (e.g., active learning, RLHF, interactive model steering, post-hoc validation/escalation, prompt-based workflows) into a single comparison view that specifies required human inputs, typical costs, risks, and failure modes (Table 1).
- We synthesize cross-cutting deployment challenges—scalability of oversight, cognitive load, trust calibration, and security/adversarial manipulation—and connect them to concrete design concerns discussed throughout the paper.
- We outline open research directions and articulate a design-oriented perspective for moving from static HITL configurations toward adaptive human–AI oversight architectures.

### 1.1. Distinct Contributions Relative to Prior HITL Surveys

In order to clarify the novelty of the manuscript with respect to previous HITL review papers, four key distinguishing factors are emphasized. First, an integrative 3D taxonomy is utilized (loop placement, interaction granularity, temporal characteristics), which allows for a more straightforward comparison across systems that may be classified under similar HITL designations but differ with respect to workload, latency tolerance, and oversight costs. Second, a wide range of application domains from healthcare to autonomous systems, cybersecurity, finance, education, and industry is synthesized under a unified framework that allows for cross-domain comparison while respecting domain-specific constraints. Third, whereas previous reviews focus primarily on the description of the various methods available for HITL design, a synthesis of the relevant trust calibration and ethical governance considerations is provided. Finally, a design-focused approach is taken that emphasizes the connections between the various method families, failure modes, and ethical considerations to configuration possibilities within a practical HITL design.

### 1.2. Systematic Review Protocol

To clarify the review methodology, we followed a PRISMA-aligned workflow for evidence identification, screening, eligibility assessment, and synthesis. The core systematic corpus used for structured synthesis comprised 134 studies; additional references were included selectively for methodological background, policy context, or illustrative domain examples.

Database search strategy.

We searched Scopus and Google Scholar for studies published between January 2018 and January 2026. The search combined HITL and domain terms, including variants of “human-in-the-loop”, “human oversight”, “human-AI collaboration”, “human-on-the-loop”, “active learning”, “RLHF”, “AI governance”, and domain qualifiers (healthcare, autonomous systems, cybersecurity, finance, education, and manufacturing). Forward/backward citation tracking was then applied to capture high-relevance studies not retrieved in the initial database query.

Inclusion/exclusion criteria.

Studies were included when they: (i) addressed explicit human involvement in AI system training, inference, supervision, or governance; (ii) reported conceptual, methodological, or empirical findings relevant to HITL design/evaluation; and (iii) were available in full text in English. Studies were excluded when they: (i) used HITL terminology without substantive human-role specification; (ii) were purely opinion/editorial pieces without analyzable technical or empirical contribution; (iii) duplicated substantially overlapping content; or (iv) fell outside the scope of AI-assisted decision systems.

Screening and selection process.

**Figure 2:**
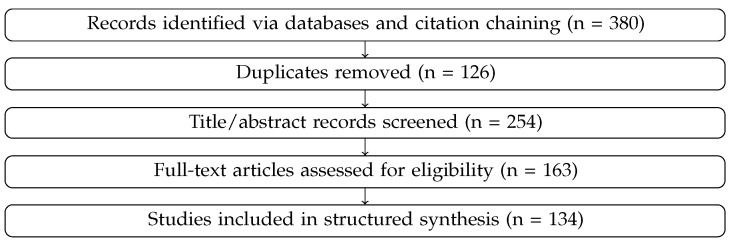
PRISMA-aligned study selection flow used for the systematic core corpus.

Screening proceeded in three stages: title/abstract review, full-text eligibility assessment, and final synthesis coding. Figure 2 summarizes the resulting flow.

## 2. Theoretical Foundations

### 2.1. Anchor Tables: Methods and Domains

To help readers navigate the HITL design space, Table 1 and Table 2 summarize (i) common HITL method families and (ii) how HITL design choices typically manifest across high-stakes application domains.

Table 1 organizes method families by required human input, indicative cost, key risks, and common failure modes; bullet-style entries in dense cells are used to improve scanability and support quick cross-row comparison.

Table 2 compares application domains using consistent headings (human oversight points, regulation/standards pressure, evaluation metrics, and pitfalls) to make differences in oversight design and deployment constraints easier to interpret.

The intellectual underpinnings of Human-in-the-Loop AI are varied, drawing upon cybernetics, cognitive science, decision theory, as well as human factors engineering. An understanding of the intellectual underpinnings of HITL AI systems provides a context for the design principles and techniques that characterize modern HITL systems. This section follows the historical path of human–machine collaboration, the philosophical underpinnings of the current research, as well as the concept of hybrid intelligence, which structures much of the current research in the field.

### 2.2. Historical Evolution

The role of human judgment within an automated system can be traced back to before the advent of modern artificial intelligence by several decades. The foundational work done by cybernetic researchers in the 1940s and 1950s provided foundational principles for feedback control that are still relevant today for human-in-the-loop (HITL) design. The work done by Norbert Wiener on human–machine systems identified the need for mechanisms to be present that enable humans to observe system behavior and act accordingly. These ideas contributed to the development of decision support systems in the 1960s and 1970s, which did not replace human decision-makers but complemented them in business management or military command scenarios [5].

The shift from decision support paradigms to interactive machine learning can be seen as a major shift in the way the role of humans is conceptualized in intelligent systems. Instead of seeing humans as mere receivers of system-suggested decisions, interactive approaches emphasize the role of humans in the process of machine learning itself. This shift is also partly driven by the understanding that many problems in reality involve tacit knowledge, contextual information, and value judgments that are difficult to formally specify [10]. The emergence of active learning algorithms in the 1990s formalized methods for systems to query human experts strategically, optimizing the use of scarce human attention and expertise.

Recent advances in deep learning have paradoxically reinforced the importance of human involvement despite dramatic improvements in automated performance. The opacity of neural network models, their susceptibility to distributional shift, and their potential for encoding harmful biases have made human oversight essential for responsible deployment [11]. The present-day focus of human-in-the-loop (HITL) studies is on both using human knowledge to improve model performance and offering effective human oversight/correction for AI systems that are integrated into complex environments. This is a process of maturity in understanding that we are not trying to minimize human involvement but are instead seeking to maximize its utilization in conjunction with machine performance based on the capabilities of each.

### 2.3. Philosophical and Cognitive Perspectives

The human-in-the-loop (HITL) design also gives rise to some fundamental issues related to the very nature of intelligence and human judgment. The proponents of human-centric views argue that some aspects of decision-making processes, such as morality and accountability, should remain exclusive to human capabilities and should not be entrusted to machines regardless of their capabilities [12]. This view draws support from phenomenological traditions that highlight the embodied and situated nature of human understanding, which differs qualitatively from computational information processing.

The bounded rationality concept, which was first proposed by Herbert A. Simon, provides a different point of view that has major implications for the design of human-in-the-loop systems. According to Simon’s theory, decision-makers are faced with information constraints, cognitive limitations, and time constraints that force them to satisfice rather than optimize. In this regard, human-in-the-loop systems have the ability to deal with vast amounts of information, identify relevant choices, and present information in a format that is cognitively friendly [12]. At the same time, designers must recognize that AI systems introduce their own forms of bounded rationality, including training data limitations, objective function misspecification, and inability to reason about situations outside their training distribution.

The question of cognitive load management is central to effective HITL design. Humans interacting with AI systems must process system outputs, maintain situational awareness, make decisions, and provide feedback, all while managing competing demands on attention and working memory [13]. Research in human factors has established that poorly designed automation can actually degrade human performance by inducing complacency, reducing skill maintenance, or overwhelming operators with alerts and information. Effective HITL architectures must therefore balance the benefits of automation against these cognitive costs, designing interactions that keep humans appropriately engaged without exceeding their information processing capacity [14].

### 2.4. Hybrid and Centaur Intelligence

The metaphor of the centaur, a mythological creature combining human and equine elements, has gained currency as a way of conceptualizing human–AI collaboration. Tang proposed the Chiron Imperative, a framework identifying six models for creating human–AI centaurs that combine the wisdom and ethical judgment of humans with the computational power of AI systems [15]. This framework emphasizes that effective collaboration requires more than simply dividing tasks between humans and machines. Instead, it calls for designing systems where human and artificial intelligence amplify each other’s capabilities in ways that neither could achieve alone.

The idea of the centaur is rooted in the domain of competitive chess, where human–computer collaborations have demonstrated capabilities beyond the capabilities of either humans or computers individually. In the context of freestyle chess tournaments, the winning teams did not comprise the individuals with the highest capabilities or the computers with the highest capabilities, but rather the teams that developed the best methods for interacting with one another. This suggests that the interface for interaction between humans and artificial intelligence systems can be as important as the capabilities of the human or the artificial intelligence system [16]. Translating this insight to other domains requires understanding the specific forms of complementarity that exist between human and machine capabilities in each application context.

The hybrid intelligence concept extends beyond task allocation to consider how human and machine learning can co-evolve over time. In this view, HITL systems are not static configurations but dynamic partnerships where both participants adapt based on their interactions [5]. Human cognition involves creating mental constructs of the capabilities and limitations of artificial intelligence systems and being able to identify when to trust the advice provided by the system or when to trust the decision-making capabilities provided by the artificial intelligence on their own. At the same time, artificial intelligence systems can be designed that mirror human desires, levels of expertise, and decision-making patterns in order to optimize the effectiveness of collaboration between humans and artificial intelligence systems [8].

### 2.5. Terminology and Loop Configurations

The proliferation of terminology for different human–AI relations is an outcome of both the diversity of the methodological approaches adopted and the lack of standardized terminology within the domain. The most common distinction is between the human-in-the-loop and human-on-the-loop relations. In the former, human participation is necessary for system functioning, often by means of active participation in decisions or authorizing system actions. In the latter case, human monitoring of system functioning is optional while allowing for possible intervention [17].

Singh and Szajnfarber proposed a more nuanced taxonomy that distinguishes Human-in-the-Loop, Human-on-the-Loop, Human-over-the-Loop, Human-under-the-Loop, and Human-along-the-Loop configurations [17]. Each configuration implies different relationships in terms of power, responsibility, and interaction rate between humans and AI systems. Human-over-the-Loop suggests a configuration where humans are in a position of power with respect to system goals and constraints. Human-under-the-Loop describes a configuration where AI systems are used for controlling or influencing human behavior. Human-along-the-Loop suggests a configuration where humans and AI systems perform related tasks in parallel with lateral interaction. To clarify the operational roles of humans and AI in HITL settings, Table 3 contrasts common loop configurations and their typical deployment contexts.

**Figure 3:**
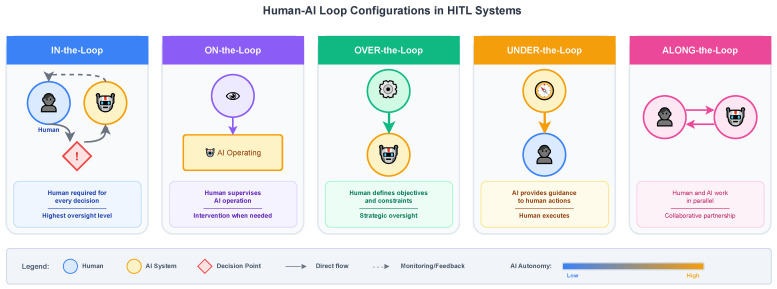
Human–AI loop configurations in Human-in-the-Loop (HITL) systems—In-the-Loop, On-the-Loop, Over-the-Loop, Under-the-Loop, and Along-the-Loop—illustrating varying degrees of human oversight and AI autonomy, from direct human participation in every decision to parallel human–AI collaboration with monitoring, strategic control, guidance, and feedback mechanisms, highlighting the flexible design space of HITL systems. Circles denote human/AI entities or states, while arrows denote information, control, and feedback flow between them.

The choice among these configurations depends on multiple factors, including the stakes involved in decisions, the reliability of AI components, regulatory requirements, and the availability of qualified human operators [6]. High-stakes applications with high harm potential usually require a Human-in-the-Loop or a Human-over-the-Loop configuration to ensure significant control. Conversely, low-stakes applications may be satisfied with a Human-on-the-Loop monitoring configuration. Understanding the different configuration options and their implications is important to the architects of human–AI systems to achieve a balance between performance, safety, and resource efficiency [18]. Figure 3 illustrates these Human–AI loop configurations, highlighting how different placements of human involvement correspond to varying levels of oversight and autonomy.

### 2.6. Conceptual Scope and Term Boundaries

In consideration of the tendency for these terms to be used interchangeably in the literature, clear scope distinctions are provided in this review. HITL AI is specified as the overarching design paradigm by which human input has operational impact on model development, deployment, supervision, or governance. Human–AI collaboration is a more general socio-technical construct that includes HITL AI but also spans configurations where humans and AI collaborate without a discernible loop-based control structure. Interactive machine learning (IML) is specified as a methodological sub-set of HITL AI that prioritizes the iterative update of models based on ongoing human interaction. Reinforcement learning from human feedback (RLHF) is specified as a particular type of HITL-based training paradigm that involves the conversion of human preference or critique into reward functions for policy optimization. Explainable AI (XAI) is specified as a supporting layer rather than a type of loop that provides benefits in terms of increased interpretability, trust calibration, or auditability without necessarily providing a basis for meaningful human control.

Consequently, the definitional hierarchy that is used in this survey is: (i) HITL AI as the overarching framework, (ii) method families such as IML and RLHF as specific instantiations of that framework, and (iii) cross-cutting enablers such as XAI and trust calibration that enable effective oversight of multiple methods and domains. This hierarchy is used uniformly in the following sections of this survey to identify: (i) the loop structure, (ii) the technical integration of human feedback, and (iii) the enablers that make oversight effective in practice.

### 2.7. Interaction Granularity and Temporal Characteristics

While loop placement describes where human authority is positioned, two additional dimensions are needed to characterize how collaboration unfolds in practice: interaction granularity and temporal characteristics. These dimensions affect annotation cost, cognitive load, latency, and safety, and therefore influence whether a HITL design remains feasible at deployment scale [13,18].

The concept of interaction granularity refers to the level and detail of human input that is expected by the system. The coarse-grained interaction model describes human input as being sparse and high-level. Examples include approval or rejection of a model’s output, escalation decisions, and quality assessment at a batch level. The medium-grained interaction model includes selective corrections, ranking or alternatives, and labeling of uncertain data in active learning. The fine-grained interaction model requires detailed human input. Examples include token-level corrections, corrections in feature attributions, trajectory guidance in reinforcement learning, and step-wise guidance in interactive generation. The more fine-grained the interaction model, the greater the possibility for precise matching and the greater the human input burden and fatigue [19,20].

For example, a coarse-grained interaction may be defined as a physician simply accepting or rejecting the model’s suggested level of urgency for a given case under AI-assisted emergency triage. On the other hand, a fine-grained interaction may require the physician to edit specific components of the model’s rationale, such as the weighting of symptoms or risk factors, providing a more detailed corrective signal at a higher temporal and cognitive cost.

The temporal properties concern the rate at which human input is integrated into the model’s behavior. Synchronous interaction requires immediate human involvement in the decision-making process (e.g., confirmation of a clinical decision prior to taking an action), while asynchronous interaction allows delayed input to be used to inform future model behavior (e.g., periodic relabeling or retrospective audit feedback). Another important distinction lies along the dimension of the rate of updates. Continuous feedback flows provide rapid adaptability to changing conditions but may cause unstable model behavior given noisy feedback, while episodic feedback occurs on a pre-scheduled review cycle to provide improved traceability of governance interventions at the expense of response speed [5,8].

There is a strong interaction between these two dimensions and the placement of the loop. For example, human-on-the-loop supervision is often used with coarse-grained and asynchronous interaction for moderate-risk operations, whereas human-in-the-loop control for high-risk operations often requires finer-grained and more synchronous interaction. Likewise, human-over-the-loop governance can use episodic temporal patterns, even for highly automated operation. This overlooks the fact that the placement of the loop is not the only relevant axis for the taxonomy.

From a design perspective, this three-dimensional taxonomy supports explicit trade-off analysis. Systems that prioritize throughput may select coarser interaction and episodic review, then add targeted synchronous checkpoints for edge cases. Systems that prioritize accountability and value alignment may adopt finer-grained interventions at selected stages while constraining interaction frequency to preserve human attention. Throughout this survey, technical methods (Section 3), application deployments (Section 5), and governance mechanisms (Section 7) are interpreted through these trade-offs to clarify why similar HITL labels can correspond to very different operational realities.

## 3. Technical Approaches

The technical underpinnings of Human-in-the-Loop AI technologies include a variety of techniques that enable significant human involvement in machine learning processes. These techniques include well-established approaches like active learning and human-in-the-loop, as well as more recent developments in reinforcement learning with human feedback and generative AI systems. This section examines the fundamental technical concepts that are integral to successful human–AI interaction, with a focus on how these techniques overcome challenges like efficiency, alignment, and reliability. Table 4 summarizes the main technical approaches used to incorporate human input in HITL AI systems, describing the underlying mechanism, the required type of human contribution, and representative studies.

### 3.1. Cross-Method Comparative Analysis and Applicability Boundaries

To enable a rich horizontal comparison between methodological classes, this paper considers the primary human-in-the-loop (HITL) techniques along six different dimensions: typical use cases, type of human involvement, interaction costs, scalability, real-time capabilities, and robustness against distribution shift/adversarial pressure. Active learning and human annotation tend to be advantageous in data-scarce domains where label quality is the primary bottleneck; these techniques offer high controllability but can become prohibitively expensive in large-scale annotation scenarios. Reinforcement learning from human feedback and preference optimization can be effective in supporting behavior alignment in generative models but suffer from increased interaction costs and require stronger countermeasures against reward hacking, preference drift, and evaluator inconsistency.

The interactive machine learning model and human guidance are best suited when iteration is possible during development, where domain specialists are readily available, allowing for rapid adaptation of local task alignment, but they are also prone to non-stationary feedback and operator variance. The post hoc validation and escalation approaches are best suited for scalability and ease of deployment, particularly when uncertainty triage is effective, but are not effective in addressing model structure issues if data or model problems are not addressed. The prompt-based human-in-the-loop model is best suited for cost-effective, rapid adaptation for generative problems but is also prone to brittleness when faced with adversarial examples and needs verification protocols for maintaining factual accuracy.

In all these dimensions, the fundamental trade-off for each method concerns not just the accuracy of the model itself, but the attention that humans have available over a period of time. Methods that demand a high level of human interaction are those that improve the accuracy of alignment while sacrificing scalability; those that require little or no human interaction heavily depend on the quality of confidence calibration, escalation, and governance. This comparative analysis is used throughout this section to highlight the strength that each method exhibits, the scope for which each method is applicable, and the measures that need to be taken for each method.

### 3.2. Active Learning and Human Annotation

Active learning represents one of the most mature and widely deployed approaches to Human-in-the-Loop machine learning. The fundamental principle underlying active learning is that machine learning algorithms can achieve better performance with fewer training examples if they are allowed to select the data from which they learn [19]. Rather than training on randomly sampled data, active learning systems identify instances where human annotation would be most informative for improving model performance. This selective approach to data labeling addresses a persistent challenge in machine learning: the high cost and limited availability of human-annotated training data.

The query strategies employed in active learning systems determine which instances are presented to human annotators. Uncertainty sampling, one of the most common strategies, selects instances for which the current model has the least confidence in its predictions [19]. Query by committee methods utilize a committee of models and select data points based on the strongest disagreement among the committee. Expected model change methods select data points that will result in the largest change in the current model. Huang et al. proposed a fast active learning method that optimizes the selection process for active learning while minimizing computational cost. This demonstrates that a well-designed algorithm can greatly improve the usability of active learning in constrained environments [21]. Each strategy embodies different assumptions about what makes an instance informative, and the choice among them depends on the specific characteristics of the learning task and the available computational resources.

The design of annotation interfaces and workflows significantly affects both the quality and efficiency of human labeling efforts. Effective annotation systems must balance the need for detailed, accurate labels against the cognitive demands placed on human annotators [34]. Research has shown that annotation quality can degrade substantially when annotators experience fatigue or when task complexity exceeds their working memory capacity. Modern annotation platforms therefore incorporate features such as adaptive task difficulty, real-time feedback on annotation consistency, and mechanisms for identifying and resolving disagreements among multiple annotators. Alla proposed an intelligent automation framework that integrates active learning with AI-driven feedback loops, enabling systems to adapt their query strategies based on annotator performance patterns [22].

The emergence of crowdsourcing platforms has expanded the scale at which human annotation can be performed while introducing new challenges related to annotator expertise and quality control. Crowdsourced annotation enables rapid collection of large labeled datasets but requires careful attention to annotator selection, training, and quality assurance [24]. Techniques such as the use of gold standard questions, inter-annotator agreement measures, and weighted aggregation of multiple annotations are helpful in maintaining the quality of labels. Wiethof et al. studied the gamification approach to boost the motivation of the annotators. Gamification can increase the quality of each annotation as well as the overall quality of all annotations by reducing the monotony of the tasks [25]. The trade-offs between expert annotation and crowdsourced labeling depend on task complexity, the availability of domain expertise, and the acceptable level of label noise for the downstream application.

A more challenging case arises when the disagreement is due to underlying ambiguity, insufficiently defined task definitions, or genuine, albeit varying, expert judgment, as opposed to error due to randomness. In these situations, majority voting can lead to the suppression of relevant, albeit minority, interpretations. Human-in-the-loop (HITL) systems can take advantage of the ability to model the annotators, for instance, through probabilistic voting, which can estimate annotator reliability and bias, or confusion matrix-based methods, which can distinguish systematic from random error. Uncertainty can be propagated downstream as soft labels or label distributions, rather than as single, discrete class targets. Operationally, the system can identify the high-disagreement instances as candidates for additional processing, for instance, through additional expert review, while low-disagreement, stable instances are left within the high-throughput annotation system. This addresses the issue of disagreement as a means to improve both the calibration of the model as well as the governance of the system, highlighting the areas of strong human agreement as well as areas of human judgment that are contestable [24,26,35].

Domain-expert annotation presents distinct challenges and opportunities compared to crowdsourced approaches. In fields such as medical imaging, legal document analysis, and scientific research, annotations require specialized knowledge that cannot be readily obtained from general crowdsourcing platforms [26]. Expert annotators can provide richer, more nuanced labels but are scarce and expensive resources. Chandler et al. examined human-in-the-loop methodologies for psychiatric applications, demonstrating how expert clinicians can be effectively integrated into machine learning workflows while respecting the constraints on their time and cognitive resources [27]. Active learning becomes particularly valuable in expert annotation contexts because it maximizes the information gained from each expert interaction. Hybrid approaches that combine expert annotation for difficult cases with crowdsourced annotation for straightforward instances can achieve favorable trade-offs between cost and quality.

Specialized annotation tasks often require custom interfaces and protocols tailored to the specific characteristics of the data and the expertise of annotators. Butler et al. developed a human-in-the-loop system for analyzing facial expression labels, addressing the particular challenges of annotating affective data where ground truth is inherently subjective and context-dependent [36]. Their work illustrates how annotation systems must be designed with careful attention to the nature of the labeling task and the cognitive processes involved in human judgment. Similarly, applications in industrial quality inspection have required annotation interfaces that present visual information in ways that support rapid and accurate defect identification by trained inspectors [23].

Recent advances in generative AI have created new possibilities for human-in-the-loop annotation workflows. Large language models can generate candidate annotations or explanations that human annotators then verify, correct, or refine [34]. This approach can substantially accelerate annotation throughput while maintaining human oversight of the final labels. Chen et al. demonstrated this framework in an educational context, developing a generative AI-based system for creating teaching materials where human educators review and refine AI-generated content [37]. The human function changes from producing annotations de novo to evaluating and editing the proposals that machines make. The empirical results show that a verification-based process can reduce the time spent in annotation while preserving or improving the quality of the annotations, provided that annotators are aware of the potential for automation bias and do not over-rely on machine recommendations.

The integration of active learning with explainable AI techniques offers promising directions for improving annotation efficiency and quality. When active learning systems can explain why a particular instance was selected for annotation, human annotators gain insight into the model’s current limitations and can provide more targeted feedback [23]. Explanations can also help annotators understand edge cases and ambiguous instances, leading to more consistent labeling decisions. Harris demonstrated how combining human-in-the-loop systems with AI fairness toolkits can help identify and mitigate biases in training data, particularly in sensitive applications such as job hiring algorithms where annotation decisions can have significant social consequences [38]. This combination of active selection, explanatory context, and fairness awareness represents a more sophisticated form of human–AI collaboration than traditional active learning approaches that treat annotation as a simple labeling task.

### 3.3. Human-in-the-Loop Reinforcement Learning

Reinforcement learning from human feedback has emerged as a powerful approach for training AI systems that align with human preferences and values. Traditional reinforcement learning relies on reward functions that specify desired behavior through numerical signals, but designing appropriate reward functions for complex tasks proves extremely difficult in practice [29]. Human-in-the-loop reinforcement learning addresses this challenge by incorporating human judgment directly into the learning process, either through explicit reward signals, demonstrations of desired behavior, or comparative preferences between alternative actions.

The most direct form of human involvement in reinforcement learning is human reward shaping, where human observers provide reward signals based on their evaluation of agent behavior. This approach has proven effective in domains where the objectives are clear to human observers but difficult to formalize mathematically [28]. In the context of autonomous driving scenarios, it is easy for humans to ascertain whether a driving action is safe and comfortable or not, even though it would be extremely challenging to define the safety and comfort criteria precisely within a reward function. The role of human rewards is to enable the learning of behaviors that are aligned with what can be considered implicit expectations.

Demonstration-based learning, also known as learning from demonstration or imitation learning, leverages human expertise by training agents to replicate observed human behavior. In this context, human experts perform tasks while the system records their actions, and the agent learns a policy that reproduces these demonstrated behaviors [29]. The method proves particularly beneficial when the behavior is difficult to articulate but easy to illustrate, a case that often arises in physical manipulation tasks, physical skills like art, and complex decision-making in dynamic environments. The quality of the learned behavior is heavily dependent on the proficiency of the human demonstrators and the number of demonstrations provided.

The idea of humans as mentors for artificial intelligence can be seen as an extension of demonstration-based learning since it enables continuous learning with human mentoring as opposed to only initial mentoring. Huang et al. introduced a framework that enables mentors to correct the behavior of an agent in real time, provide additional demonstrations for complex scenarios, and adjust learning based on performance [28]. This model of mentorship recognizes that effective learning processes are often realized through adaptive guidance that is responsive to the learner’s current capabilities and specific issues that emerge during training processes. The role of a mentor is one that involves less supervisory control than that of teleoperation but offers more feedback than that of demonstration collection.

Preference-based reinforcement learning represents a particularly influential approach that has enabled significant advances in language model alignment. Rather than providing explicit rewards or demonstrations, humans express preferences between pairs of agent behaviors, indicating which outcome they prefer [30]. Such preference comparison is subsequently used for training a reward model that represents human values, with the learned reward model guiding the agent learning. The preference-based approach relieves human evaluators from cognitive burdens by replacing absolute judgments with relative comparison, which humans are more likely to do uniformly.

The operational risks involved in reinforcement learning from human feedback are significant and must be addressed as first-class design considerations rather than auxiliary caveats. Reward models carry the risk of encoding evaluator bias, discounting minority opinions, and being vulnerable to reward hacking or specification gaming if policies over-optimize proxy reward signals. There are also risks of preference drift over time, brittleness in the face of distribution shift, and safety regressions that are only discovered post-deployment via interactions. These are the reasons for the importance of ongoing auditing, red teaming, and rollback planning in RLHF pipelines, in addition to optimization (see Table 1).

Another important difference from a technical standpoint is whether reinforcement learning from human feedback (RLHF) is conducted online or offline. Offline RLHF makes use of a dataset, which can improve the reproducibility of the system as well as pre-deployment governance, although the system may not capture rare threats as well as online RLHF. On the other hand, online RLHF can learn from interaction with users, which can improve the system’s ability to correct its own behavior, although the system may become more susceptible to adversarial attacks, feedback loops, as well as rapid policy change without human intervention. Thus, online RLHF is more difficult to integrate with a high-assurance validation approach than offline RLHF [29,30].

Safety considerations are paramount in human-in-the-loop reinforcement learning, particularly for applications in autonomous systems and robotics. Learning agents may explore dangerous actions during training, and the consequences of unsafe behavior can be severe in physical environments [28]. Human involvement in these environments has several safety-related functions: detection and prevention of potential hazardous actions before execution, provision of corrective feedback upon the occurrence of hazardous actions, and specification of safety constraints that restrict the action set for the agent. The design of human–AI interfaces for safety-critical reinforcement learning agents should allow for prompt human involvement while disturbing the learning process as little as possible.

The application of human-in-the-loop reinforcement learning to autonomous driving has produced substantial research contributions and practical systems. Autonomous vehicles must navigate complex traffic environments while satisfying multiple objectives including safety, efficiency, passenger comfort, and compliance with traffic rules [29]. Human-in-the-loop approaches enable these systems to learn driving behaviors that satisfy human expectations across these multiple dimensions. Real-time human guidance during training can help agents learn appropriate responses to rare but important situations that might be underrepresented in demonstration data or difficult to specify through reward engineering [28]. Ahmad examined the broader question of how human-in-the-loop AI models can support trustworthy autonomous driving systems, emphasizing the importance of maintaining meaningful human oversight even as vehicle automation capabilities increase [39].

Control room and industrial applications present distinctive requirements for human-in-the-loop reinforcement learning. Operators in process control environments must manage complex systems with multiple interacting variables, competing objectives, and significant consequences for errors [40]. The reinforcement learning agents can assist the operators in suggesting actions, predicting outcomes, or identifying anomalies; yet, the decision-making prerogative lies in the hands of the operators. Research studies in this area have investigated the cognitive states of the operators, which include fatigue, workload, and trust, that affect the effectiveness of human–AI collaboration. Emmanouilidis et al. researched the integration of human-in-the-loop AI systems into production environments, which pinpointed key factors that affect the effectiveness of the integration [41].

Apart from industrial control systems, human-in-the-loop reinforcement learning has also found some applications in building management systems and environmental control systems. Liang et al. proposed a human-in-the-loop AI system for HVAC management that meets both efficiency and comfort requirements. This shows that reinforcement learning agents can be used to meet human requirements that differ from person to person [42]. This application illustrates how human feedback can guide learning in domains where objectives are inherently subjective and where automated systems must adapt to diverse user preferences.

Adaptive learning systems in education represent another promising application domain for human-in-the-loop reinforcement learning. Tarun et al. explored how generative AI combined with human-in-the-loop feedback can create personalized learning experiences that adapt to individual student needs [43]. In these systems, human educators are used to provide feedback, which is used to guide the AI system. The reinforcement learning framework is used for the refinement of educational interventions, which is informed by the learning outcomes as well as the educators’ feedback.

Swarm intelligence approaches offer an alternative example for incorporating human input into collective AI systems. Rosenberg’s work on artificial swarm intelligence demonstrated that groups of humans connected through real-time feedback systems can function as unified intelligent systems that outperform both individual humans and traditional AI approaches on certain tasks [44]. This approach turns the traditional human-in-the-loop concept on its head, as it involves the incorporation of artificial intelligence into collective human processes, as opposed to incorporating humans into artificial intelligence systems. This results in a hybrid swarms concept, which combines human intuition and understanding with machine-based aggregation and coordination.

### 3.4. Generative AI with Human-in-the-Loop Feedback

The accelerated development of generative AI systems, with a focus on large language models, has enabled a number of emerging paradigms for human-in-the-loop interaction that are quite different from traditional machine learning approaches. These generative models are capable of creating text, code, images, and other media at a quality that approaches or rivals human levels, yet they require human intervention to ensure that they are correct with respect to user intent, factual correctness, and ethical appropriateness [31]. The human role in generative AI systems encompasses prompt design, output evaluation, iterative refinement, and ongoing monitoring of system behavior across diverse use cases.

Prompt engineering is a skill that has emerged as a key competence for successful human–AI collaboration with large language models, where the quality and precision of prompts play a significant role in determining their relevance, accuracy, and utility. Ranade et al. showed that rhetorical strategies can be applied in a systematic way for prompt engineering, conceptualizing the interaction between humans and AI as a communicative process for which principles of effective discourse are well established [31]. This perspective reframes prompt engineering from ad hoc experimentation to a principled practice grounded in communication theory. Effective prompts must convey not only the desired task but also relevant context, constraints, output format preferences, and quality criteria.

However, the quality of the response does not address the underlying structural failure modes of the generative model. The issues of hallucination, factual inconsistency across generated responses, and stability with respect to minor changes in the prompts continue to be core technical risks with HITL. The reasons for these risks are that the responses generated are fluent and plausible even when they are incorrect. This makes human over-trust a significant risk in a high-throughput scenario. This essentially means that HITL-style governance must treat generated responses as statements that must be verified rather than as texts that must be rewritten [32,45].

In a technically sound human-in-the-loop (HITL) pipeline, generation and verification are kept decoupled as a matter of course. In most cases, standard security measures include retrieval-grounded generation, citation or evidence fields, and consistency checks among various model versions. In addition, in a scaled-up environment, organizations may employ a system of triage, where generated content is categorized into risk levels, with low-risk content possibly subjected to spot checks, whereas high-priority content, such as medical, legal, or financial, may require a more complex system of structured review and sign-off accountability. In a system where verification is not layered in such a manner, it may become a bottleneck, with human verifiers reverting to superficial approval-based behaviors that are not adequate for infrequent yet significant errors [46,47,48].

The iterative refinement of generative AI outputs represents a distinctive form of human-in-the-loop interaction. Unlike traditional machine learning where human input primarily occurs during training, generative AI systems enable continuous human feedback during inference [32]. There is a capacity for users to judge the content generated, recognize areas for improvement, and offer remedial advice that can guide the subsequent content. This form of dialogue allows human users to guide the content towards the desired form without the need to specify the requirements a priori. The model is more similar to co-editing than supervising, as the human user and the AI system work together to create content through a series of iterations.

Chain-of-thought prompting and related techniques have demonstrated that encouraging language models to articulate intermediate reasoning steps can substantially improve performance on complex tasks. Atkinson extended this approach through chain-of-code prompting, which integrates human validation at key points in multi-step reasoning processes [33]. Human evaluators can authenticate intermediate conclusions, correct inaccuracies in the reasoning process, and provide guidance when the model is uncertain. This nested human-in-the-loop model improves the reliability of processing complex tasks by combining the generative capabilities of language models with human judgment at the point of decision-making. Fu et al. extended the concept of combining language models with human judgment by incorporating non-monotonic logical reasoning, thereby creating assistive AI agents with more robust reasoning capabilities under uncertain situations [49].

The application of human-in-the-loop generative AI to professional domains has produced systems that augment expert capabilities while maintaining appropriate oversight. Bui examined the use of generative AI with human oversight for patent law applications, including AI-assisted drafting, prior art search, and multimodal intellectual property protection [50]. These applications require high accuracy and must satisfy strict professional standards, making human validation essential despite the capabilities of underlying AI systems. Yuan et al. developed Alpha-GPT 2.0, a human-in-the-loop system for quantitative investment that combines language model capabilities with human trader expertise to generate and refine investment strategies [51]. In both cases, the human role extends beyond simple approval to include substantive evaluation of AI-generated content against domain-specific criteria.

The healthcare applications of generative AI pose unique challenges in human-in-the-loop design due to the potential impact of erroneous outputs and the need for accountability. Fahad and Huang suggested a framework for continuous validation in healthcare applications of generative AI outputs. They emphasize that human involvement should be an integral part of the workflow and not just at the end stages [32]. Their framework addresses the issue of maintaining diligent human review in the face of the usual high-quality output of AI systems while also acknowledging that occasional errors can have significant consequences in clinical settings. The construction of a robust human review for medical generative AI needs to take into consideration the cognitive burden on clinicians, time constraints, and the need to prevent the degradation of clinical skills that can result from over-reliance on AI.

Financial services represent another domain where generative AI is being deployed with human-in-the-loop safeguards. Singh proposed a five-step governance framework for generative AI in banking that operationalizes trust through structured human oversight at multiple stages [48]. The model recognizes that regulatory demands, reputation, and fiduciary duty require a high level of human oversight for AI outputs in a financial setting. Anniciello et al. studied human-in-the-loop generative AI for insurance decision support. The authors created an explainable system that provides justifications for AI recommendations [45].

The balance between the efficiency of automated systems and the effectiveness of human oversight is a key challenge for the deployment of generative artificial intelligence systems. Verma examined if generative AI could be used as a substitute for human-in-the-loop methods in urban design research. He found that although generative AI could speed up some tasks, human judgment was necessary for evaluating the quality and contextual appropriateness of the design [52]. This finding echoes broader concerns about maintaining meaningful human engagement as AI capabilities improve. Effective human-in-the-loop generative AI systems must be designed to keep humans cognitively engaged and capable of identifying AI errors, rather than reducing humans to passive approvers of AI outputs.

Content generation at scale introduces additional considerations for human-in-the-loop workflows. Nuotio investigated the impact of generative AI on journalistic processes, examining how human-in-the-loop approaches can maintain editorial standards while leveraging AI capabilities for content production [46]. Organizational factors that are relevant for successful integration were identified, e.g., clear job definitions, provision of training for human reviewers, and quality assurance approaches that are adapted for AI-based workflows. Kolagani and Vuppala examined related aspects in the context of enterprise customer services, proposing a hybrid approach for balancing efficiency with human oversight for quality maintenance in these services [47].

### 3.5. Explainability, Interpretability, and Trust

The ability of humans to comprehend, evaluate, and correctly depend on AI systems is significantly dependent on the explainability of AI systems. Explainable AI is a term that comprises various methods that make AI model behavior comprehensible to humans, thus aiding them in decision-making based on when to trust AI suggestions and when not to [4]. Without adequate explainability, human-in-the-loop oversight becomes superficial, as humans cannot meaningfully evaluate outputs they do not understand. The development of explainable AI methods is therefore not merely a technical convenience but a prerequisite for effective human–AI collaboration.

The difference between interpretability and explainability, although sometimes fuzzy in practice, implies a number of differences regarding the way an AI system can be made understandable. Interpretability is derived from the intrinsic understandability of the system based on structural properties such as decision trees, rule-based systems, or linear models with a reduced number of features. Conversely, explainable AI refers to methods that provide explanations for models that are not intrinsically interpretable, such as deep neural networks [53]. In the study by Assadi & Safaei, interpretable artificial intelligence is discussed in the context of product recommendation systems. This demonstrates that the effectiveness of incorporating human feedback into the loop is increased when users are able to grasp the rationale behind the decision made by the system. Both methods are intended for improving human understanding; however, there are clear distinctions between them.

Factual explanations describe the features or patterns that led to a particular AI output, while counterfactual explanations describe what would need to change for the output to be different. Ibrahim et al. conducted an algorithm-in-the-loop analysis comparing these explanation types, finding that their effectiveness depends on the decision context and the expertise of human users [54]. Counterfactual explanations proved particularly valuable for helping users understand decision boundaries and identify actionable changes. The choice between explanation types should be guided by the specific needs of human decision-makers and the characteristics of the decisions they face.

Trust calibration represents a critical challenge in human–AI systems where humans must learn to rely appropriately on AI capabilities. Both over-trust and under-trust can compromise system performance: over-trust leads humans to accept AI errors uncritically, while under-trust causes humans to reject valid AI recommendations [20]. In practice, adoption is strongest when users experience consistently calibrated trust because they view the system as both useful and safe enough to incorporate into routine workflows. Tsiakas and Murray-Rust explored how explainable AI can help humans develop appropriate trust by providing insight into AI reasoning processes and limitations. Their work emphasizes that trust should not be unconditional but calibrated to the actual reliability of AI systems across different situations and task types.

The cognitive alignment between AI explanations and human mental models significantly affects whether explanations actually improve human decision-making. Explanations that are technically accurate but do not match how humans think about a problem may fail to improve understanding or may even introduce confusion [13]. Kotsiopoulos et al. examined this issue in industrial defect recognition, developing explanations designed to align with the cognitive mechanisms that expert inspectors use when evaluating product quality. Their approach illustrates the importance of user-centered design in explainable AI, where explanation methods must be tailored to the knowledge and reasoning patterns of intended users.

The affective dimensions of human–AI interaction influence how explanations are received and whether they achieve their intended effects. Charoenrat developed an affective and explainable AI-driven model for adaptive learning that considers learner emotional states alongside cognitive factors [14]. The current research recognizes that human interactions with AI systems are not just rational in nature, as human responses to AI systems are also subject to emotional responses to the AI system’s behavior, explanations provided, and the interactive nature of the AI system. Explainable AI systems that consider affective factors may help to achieve more effective human–AI collaborations compared to AI systems that are designed based on cognitive models of human users.

The practical implementation of explainable AI in human-in-the-loop systems requires careful attention to explanation timing, format, and level of detail. Explanations that interrupt workflow, require excessive cognitive effort to process, or provide irrelevant detail can reduce rather than enhance human performance [4]. Effective explanation interfaces must balance completeness against usability, providing sufficient information for informed decisions without overwhelming users. Research on explanation design has identified principles such as progressive disclosure, where users can access additional detail on demand, and contrastive explanation, where systems highlight differences from typical cases rather than exhaustively describing all features. Table 5 summarizes trust calibration states in human–AI interaction, outlining their defining characteristics, associated risks, and practical interventions for achieving appropriate reliance.

The relationship between explainability and human learning creates opportunities for AI systems that not only support individual decisions but also help humans develop expertise over time. When explanations reveal the patterns and relationships that underlie AI predictions, humans can internalize this knowledge and apply it in situations where AI assistance is unavailable [20]. This role of explainable AI, as part of the educational goals, points to the design strategy that places human learning as a priority alongside prompt decision support. The systems developed for the achievement of these purposes may create more valuable systems for the future, as they improve human capabilities, not reliance on AI systems.

### 3.6. Trust Calibration and Human–AI Interaction Failures

The effectiveness of human-in-the-loop systems depends fundamentally on whether humans can develop and maintain appropriate levels of trust in AI components. Trust calibration refers to the alignment between a user’s confidence in an AI system and the system’s actual reliability [11]. When trust is well-calibrated, people are generally able to trust AI recommendations in cases where the system performs well, and use their own judgment in cases where the system tends to perform poorly. The challenge in achieving well-calibrated trust lies in the need for people to build accurate mental models of how well AI systems perform in a wide range of cases.

Over-trust occurs when humans place excessive confidence in AI systems, leading them to accept erroneous outputs without adequate scrutiny. Agudo et al. conducted empirical studies examining how AI errors propagate through human-in-the-loop processes, finding that humans often fail to detect and correct AI mistakes even when they have the knowledge and ability to do so [11]. This is sometimes termed automation bias or even automation complacency, and this is a major risk when AI is used in situations where errors could have serious consequences. The study also revealed that the rate of error detection reduces when individuals adapt to high accuracy levels of AI, which means that AI’s success could be its own failure in terms of requiring human oversight.

Under-trust presents the opposite problem, where humans discount valid AI recommendations due to skepticism, unfamiliarity, or negative prior experiences. Baroni et al. developed the AI-TAM model to investigate factors affecting user acceptance and collaborative intention in human-in-the-loop applications [56]. In their study, they were able to identify several determinants of trust, such as perceived usefulness, perceived ease of use, and social influence, which clearly shows that trust development involves a rational assessment of system capabilities as well as contextual factors. Under-trust may cause humans to turn away from AI assistance in cases where the performance of AI systems is significantly better than human judgment alone.

The dynamics of trust development over extended interaction periods introduce additional complexity. Lopes conducted studies on operator fatigue, trust, and workload demand in human-in-the-loop AI-enabled drone systems, revealing how trust evolves as operators gain experience and as their cognitive resources become depleted [57]. The initial level of trust, whether high or low, has a tendency to set a foundation for future trust evaluations. As such, interactions with AI at the onset have a strong impact. Fatigue was shown to affect the calibration of trust by reducing cognitive resources for monitoring and evaluation. The influence of individual differences on trust calibration has received increasing research attention. Dores Cruz et al. demonstrated that political preferences can compromise human-in-the-loop oversight of AI, with individuals showing systematic biases in how they evaluate AI outputs depending on whether those outputs align with their prior beliefs [58]. This result carries significant implications for applications in which AI systems are involved in discussions about politically or socially contested issues, suggesting that a variety of oversight bodies may be necessary in order to combat individual biases. In a more general sense, the present study underscores that trust in AI systems cannot be accounted for by system-related factors alone, but is shaped by what people believe, value, and cognitively tend toward in their interactions with others.

The issue of transparency in regard to the boundaries of artificial intelligence is one of the ways of building calibrated trust, but the relationship between transparency and calibrated trust is not immediately clear. Brooks argues that it is important to maintain proper expectations of artificial intelligence in order to enable effective cooperation between humans and artificial intelligence, but this should not be done in an optimistic or dismissively skeptical way [55]. Communicating uncertainty and limitations can help in the development of accurate mental models in humans; however, over-hedging can create a lack of confidence in the face of valuable assistance from an artificial intelligence system. Transparency that is effective requires calibration in the communication of limitations.

System failure and error recovery mechanisms can be identified as critical junctures for trust calibration. The actions taken by the AI system during such failure and its potential to help in the recovery from human errors can impact the overall level of trust. Alpay and Alpay examined the deficient human-in-the-loop oversight mechanisms in sophisticated AI systems and identified patterns that occur in such failure scenarios [59]. Their results show that trust violations resulting from unforeseen system failures are difficult to mitigate, especially when humans are not provided with clear explanations for the reasons behind these unforeseen system failures. Designing for graceful degradation can aid in ensuring trust levels are maintained in spite of unforeseen system failures in AI systems.

The organizational context in which human–AI collaboration occurs shapes trust dynamics in ways that extend beyond individual user–system interactions. James examined human-in-the-loop architectures for trustworthy AI planning in mission-critical business intelligence systems, emphasizing how organizational structures, accountability mechanisms, and cultural factors influence whether humans exercise meaningful oversight [60]. In an organizational setting, trust exists at various levels: individuals need to trust the AI system, individuals need to be trusted by the organization to provide adequate oversight, and the organization needs to trust that the overall human–AI system meets performance and safety requirements. Disalignment at these different levels can create oversight issues even if all levels appear to be functioning correctly in isolation.

Responsibility attribution in human–AI systems creates complex dynamics that affect trust and oversight behavior. When errors occur in collaborative human–AI processes, questions arise about whether responsibility lies with the AI system, the human operator, the system designers, or the organization that deployed the system [61]. Mellamphy discusses how different understandings of the relationship between humans and artificial intelligence, including humanistic and posthumanist understandings, imply different understandings of responsibility. The unclear sense of responsibility can lead to unwise human intervention due to a sense of unaccountability for artificial intelligence errors or can lead to obstructive behavior due to a sense of blame for uncontrollable artificial intelligence errors.

The scapegoat-in-the-loop concept captures situations where humans are nominally included in AI systems primarily to absorb responsibility rather than to provide meaningful oversight [61]. In these configurations, human involvement may satisfy legal or regulatory requirements without actually improving system safety or performance. Ottun and Flores conducted a review of human oversight and human-in-the-loop approaches, identifying characteristics that distinguish meaningful oversight from superficial compliance [2]. Meaningful oversight requires that humans have sufficient information, time, expertise, and authority to evaluate and override AI decisions, conditions that are not always met in practice despite nominal human-in-the-loop designs.

Adaptive methods for trust calibration attempt to control the behavior of a system by responding to patterns of trust from humans. In other words, rather than presenting the results of the AI systems in a uniform manner, it is possible for the presentation of the results or recommendations to be adjusted based on the trustworthiness of the results or the patterns of trust from individual users. This was demonstrated by Cho et al. in a wearable sensor for thermal comfort control [62]. Such adaptive approaches can help correct both over-trust and under-trust by providing stronger endorsements when AI confidence is high and more hedged recommendations when uncertainty is elevated.

The long-term sustainability of human-in-the-loop oversight requires attention to skill maintenance and engagement. When AI systems perform well consistently, human operators may experience skill decay in the tasks that AI has assumed, reducing their ability to detect errors or take over when AI systems fail [57]. Concurrently, a decrease in the cadence of substantive intervention opportunities could also foster boredom, which would further impair the quality of oversight. The development of human-in-the-loop systems for sustainable operation requires the intentional preservation of human skills and engagement, which could be done through training exercises, task allocations, or system designs that sustain substantive human involvement even when AI automation could be used for autonomous performance of tasks.

## 4. Fairness, Bias, and Value Alignment

The use of artificial intelligence in environments that affect human well-being creates essential issues in terms of fairness and bias and the relationship between algorithmic decision processes and human values. Human-in-the-loop techniques offer a means to address these issues by incorporating human judgment in processes for detecting bias, determining fairness, and identifying human values. The section examines the potential for human involvement in the development and use of AI that is equitable and just for individuals and groups while being sensitive to different stakeholder perspectives on fairness.

### 4.1. Human-in-the-Loop Fairness

The concept of fairness in AI systems defies a technical description since different stakeholders have different and sometimes conflicting ideas about what is fair. Research by Nakao et al. was instrumental in laying the groundwork for involving end users in interactive human-in-the-loop AI fairness and showing that end users can provide valuable information for fairness criteria that experts might overlook [35]. The study also demonstrates that the assessment of fairness can be improved by considering diverse viewpoints and that interactive systems can play an important role in enabling the participation of non-technical stakeholders in decisions related to fairness. The results also reveal significant differences between user groups regarding their perceptions of fairness, which suggests that fairness metrics may not be sufficient to address the fairness concerns that are most important to users.

Participatory approaches to AI fairness seek to incorporate stakeholder perspectives throughout the system development lifecycle rather than treating fairness as a post-hoc evaluation criterion. Taka et al. developed methods for integrating stakeholder feedback to incorporate fairness perspectives in responsible AI development [63]. This approach recognizes that fairness concerns are rooted in social contexts and cannot be fully specified by system developers in a vacuum. By providing formal channels through which stakeholders can contribute, participatory fairness approaches aim to uncover concerns that would not be visible to system developers and ensure that the legitimacy of these systems is established among these social groups.

Crowdsourcing offers one mechanism for gathering diverse perspectives on AI fairness at scale. Nakao examined how crowdworkers’ characteristics and the framing of fairness metrics affect perceptions of AI fairness, finding that demographic factors, personal experiences, and the specific way fairness questions are posed all influence judgments [35]. This study also highlights the complexity involved in aggregating fairness judgments from diverse populations and the need for careful consideration in the process. Though the use of crowdsourced evaluations for fairness can be useful in augmenting the results obtained from experts by understanding the population’s perspective on the system’s behavior, it is important that the crowdsourced samples be representative.

The transition from reactive fairness auditing to proactive fairness-aware design represents an important evolution in human-in-the-loop fairness research. Griffen and Owens proposed moving from traditional human-in-the-loop models to participatory systems of governance for AI in healthcare, arguing that meaningful fairness requires ongoing stakeholder engagement rather than one-time consultation [64]. The model that they have created conceptualizes the affected communities as collaborators in AI governance rather than merely being subjected to AI systems. There is a need for greater human engagement in AI governance than in the usual oversight mechanisms. There is a possibility that the values of the community can be aligned more closely with AI systems.

### 4.2. Bias Detection and Mitigation

Algorithmic bias may result from various factors like biased data, incorrect assumptions of the model, and evaluation metrics that do not correctly measure the differential impact. Human-in-the-loop strategies for algorithmic bias detection employ human judgment to detect biased outcomes that may be missed by automated metrics. The strategies also help to determine the difference between unfairness and disparities. Harris proved that the integration of human-in-the-loop systems with artificial intelligence fairness toolkits may help mitigate age bias in employment hiring algorithmic systems [38]. The research found that human involvement improved both the detection of bias and the development of effective remediation strategies.

Employment and hiring represent high-stakes domains where algorithmic bias can have severe consequences for individuals and where human oversight is particularly important. Neupane investigated algorithmic justice in AI and machine learning-enabled talent acquisition systems, examining how human-in-the-loop approaches can identify and address discrimination in automated hiring [65]. The study shows how the hiring algorithms can reflect biases related to age, gender, educational backgrounds, and other protected characteristics. These biases are often not immediately apparent by analyzing the results of the algorithms. With proper training, humans can recognize disparate treatment patterns that may not be immediately apparent through statistical analysis.

Algamaty developed approaches for fair and transparent AI in hiring that combine resume–job matching with bias mitigation and human-in-the-loop auditing [66]. The framework also incorporates several checkpoints where human evaluators rate system recommendations for possible bias before decisions are made on candidates. The hierarchical system recognizes that bias can be introduced during various stages of the hiring process, and effective mitigation of this issue requires consideration of each of these stages. The human auditing mechanism is intended not only to pinpoint specific instances of biased recommendations but also to create feedback that can improve system performance over time.

Content moderation and online platform governance present distinctive challenges for bias detection where the boundaries between harmful content and protected speech are contested and context-dependent. Sheombar examined fallacies in online hate speech detection, revealing how both AI systems and human moderators can exhibit biases in identifying fringe hate speech [67]. The study shows that human-in-the-loop configurations can both reinforce or reduce the effects of bias in AI systems depending on the way human review is structured. For effective mitigation of bias in content moderation systems, it is essential that the biases present in human evaluators be taken into account along with the methods used for measuring alignment between human and AI bias so that unfair outcomes are not allowed to occur.

The issue of mitigating biases does not only apply to individual decisions but also to the system itself, which is a result of multiple decisions made using AI-assisted decisions. Joseph & Yakubu studied human-in-the-loop decisions for various decision-sensitive domains like education and the non-profit sector. They found that while biases are small individually, their aggregated effect can be substantial [68]. Their study also emphasizes the need to monitor aggregate outcomes as well as decisions made by individuals, and it suggests that feedback mechanisms that point to patterns of disparate impact need to be created. Relying solely on human oversight to review decisions made on a case-by-case basis may not be sufficient to point to biases that only become apparent when outcomes are reviewed on a wide range of decisions.

The above studies collectively suggest that human oversight is not an inherently bias-reducing mechanism; rather, it is contingent upon design or governance decisions. Oversight is seen to be beneficial to bias mitigation to the extent that humans are provided with clear decision support criteria, fairness diagnostics, and sufficient time for review. Additionally, review bodies are better positioned to mitigate biases when they are diverse rather than representative of a single institution’s perspective. In such cases, humans are capable of detecting harms that are not captured by aggregate metrics of models and are able to take corrective actions [38,66].

On the other hand, oversight can be unproductive when human reviewers face high-throughput pressure, vague guidance, or unclear definitions of policies. In such cases, reviewers can fall back on automation biases, replicate existing social biases, or normalize existing biases as “reasonable” outcomes. Such unproductive outcomes have been observed in Sheombar’s study on hate speech moderation, where both human and model biases can “reinforce” each other [67]. A coherent HITL bias-mitigation strategy therefore requires dual-level evaluation: case-level review quality and outcome-level disparity monitoring over time, with escalation triggers when the two diverge [68].

### 4.3. Value Alignment and Accountability

The challenge of aligning artificial intelligence (AI) systems with human values is not only related to fairness but also extends to other issues related to the goals that AI systems should pursue, as well as conflicts that may arise between these values. Chen et al. developed methodologies for creating ethical AI systems based on human-in-the-loop approaches, arguing that value alignment requires ongoing human involvement, not just initial goal specification [69]. Their model recognizes that human values are complex, situation-dependent, and sometimes internally contradictory, requiring means of human judgment to control system behavior when algorithmic methods are insufficient. Chen also examined the practicality and rationality of human-in-the-loop methods for AI value alignment, including situations where human-in-the-loop methods improve value alignment and situations where they create additional complexities [70].

Regulatory frameworks increasingly mandate human oversight for AI systems that affect fundamental rights and safety. Middleton et al. examined trust, regulation, and human-in-the-loop AI within the European region, analyzing how regulatory requirements shape the design and deployment of AI systems [71]. The study revealed the tension between the need for human oversight and the limits of human attention and expertise. The study by Constantino focused on the accountability issues that arise from the implementation of Article 14 in the EU AI Act, which calls for human oversight in high-risk AI systems in public administration [72]. The analysis revealed ambiguities in how oversight requirements should be implemented and questions about whether mandated human involvement actually improves outcomes or merely shifts liability.

The attribution of responsibility when AI-assisted decisions cause harm remains contested and has significant implications for accountability and governance. Ranisch examined the phenomenon of scapegoat-in-the-loop configurations in medical AI, where human involvement may serve primarily to absorb responsibility rather than to improve decision quality [73]. In that paper, concerns are raised about human-in-the-loop systems that satisfy formal accountability requirements yet do not provide actual oversight. In a study on the irreducibility of consciousness in human intelligence and its implications for accountability in artificial intelligence, Samarawickrama discussed how some aspects of moral responsibility cannot be handed over to a system, no matter how advanced it is [74]. These philosophical considerations inform debates about the appropriate scope of AI autonomy and the conditions under which human oversight is genuinely necessary.

Ethical frameworks from diverse philosophical traditions offer resources for thinking about human–AI relationships and value alignment. Liu examined human-in-the-loop ethical AI for care robots through the lens of Confucian virtue ethics, demonstrating how non-Western ethical traditions can inform the design of AI systems that support human flourishing [75]. Tang proposed the Chiron Imperative as a framework for creating wise and just AI–human centaurs, drawing on classical concepts of practical wisdom to guide human–AI collaboration [15]. These cross-cultural and historically informed approaches expand the conceptual resources available for addressing value alignment and highlight the importance of diverse perspectives in shaping AI ethics.

Organizational and sectoral contexts shape how value alignment and accountability are operationalized in practice. Singh developed a governance framework for generative AI in banking that operationalizes trust through structured human-in-the-loop oversight [48]. The framework addresses the particular regulatory requirements and risk considerations of financial services while providing a model that could be adapted to other sectors. Joshi and Vaidya examined responsible AI adoption in small and medium enterprises, proposing frameworks that account for the resource constraints that smaller organizations face in implementing meaningful human oversight [76].

Privacy considerations interact with human-in-the-loop design in complex ways that affect both the feasibility and the ethics of human oversight. Rivadeneira et al. developed a unified privacy-preserving model for human-in-the-loop cyber–physical systems, addressing the challenge of maintaining human oversight while protecting sensitive data [77]. Their approach demonstrates that privacy and oversight need not be in fundamental tension but require careful technical design to achieve simultaneously. Anthuvan et al. examined human–AI collaboration in academic writing, developing the Scholarly HI-AI Loop Framework for ethical knowledge production that addresses questions of attribution, integrity, and accountability in AI-assisted research [8]. As AI systems become more capable of generating content that could be mistaken for human work, questions of transparency and appropriate disclosure become increasingly important for maintaining trust in knowledge production institutions. Table 6 summarizes key application domains of human-in-the-loop (HITL) AI, highlighting typical loop configurations, risk levels, and domain-specific challenges.

## 5. Applications in High-Stakes Domains

**Figure 4:**
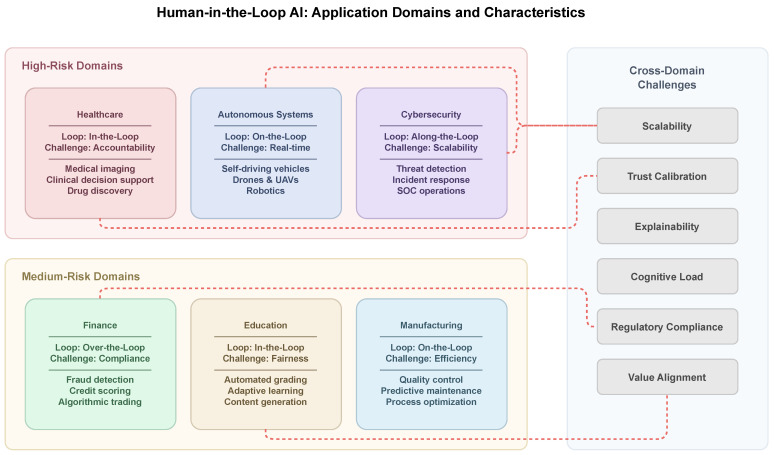
Application domains and characteristic challenges of Human-in-the-Loop (HITL) AI systems, grouping high- and medium-risk sectors (e.g., healthcare, autonomous systems, cybersecurity, finance, education, and manufacturing) by typical loop configurations and highlighting cross-domain challenges such as scalability, trust calibration, explainability, cognitive load, regulatory compliance, and value alignment that shape HITL design and deployment.

The principles and methodologies of human-in-the-loop artificial intelligence are best implemented in areas that have significant implications for human well-being, safety, and human rights. Such areas as healthcare, autonomous systems, cybersecurity, etc., are the most motivating factors for human-in-the-loop artificial intelligence and simultaneously the most challenging areas for the implementation of human–AI cooperation. This section will discuss the adaptation of human-in-the-loop artificial intelligence to the requirements of these areas. Figure 4 provides an overview of these application domains, highlighting their associated risk levels, Human–AI loop configurations, and characteristic challenges.

With respect to the operational functions, human-in-the-loop feedback processes demonstrate a range of domain-specific variation, from primarily supporting clinical validation and judgment context in the health domain, to supervisory intervention and edge case correction in the autonomous systems domain, to threat triage and decision making under uncertainty in the cybersecurity domain, to supporting compliance review and fairness/accountability assessment in the finance domain, to moderating AI output against rubric-based pedagogical criteria in the education domain, and finally, to supporting defect adjudication and adaptation processes in the manufacturing domain. This range of domain-specific variation implies that the effectiveness of HITL processes may not necessarily be related to the human element, but rather to the alignment of the feedback processes with the primary risk profile of the domain.

### 5.1. Healthcare and Life Sciences

The medical applications of AI in healthcare support a human-in-the-loop approach since decision-making in healthcare has a direct impact on health and the accountability requirements in healthcare mandate that only qualified individuals be responsible for healthcare. Bakken highlights the need for human involvement in health AI by arguing that the complexity of clinical reasoning and decision-making, as well as the relevance of the context in which patients receive healthcare and the ethical requirements in healthcare decision-making, requires involvement in AI [78]. This perspective reflects broader consensus in medical informatics that AI should augment rather than replace clinical judgment, with systems designed to support rather than supplant the expertise of healthcare professionals.

Medical imaging represents one of the most active areas for HITL AI development in healthcare. Yu et al. developed PI-RADSAI, a human-in-the-loop model for prostate cancer diagnosis based on MRI that integrates radiologist expertise with machine learning capabilities [26]. The system presents AI-generated assessments to radiologists who can confirm, modify, or reject the automated analysis based on their clinical judgment and additional patient information not available to the algorithm. Wu et al. demonstrated AI-accelerated structuring of radiology reports with human oversight, showing how AI can reduce documentation burden while maintaining the accuracy and completeness that clinical communication requires [88]. These applications illustrate the pattern of AI handling routine processing while humans focus on interpretation, verification, and communication.

Neurological applications have demonstrated the potential for HITL systems to achieve performance that generalizes across diverse clinical settings. Yang et al. developed a human-in-the-loop AI system for clinical seizure recognition that achieved continental generalization, maintaining diagnostic accuracy across patient populations in different healthcare systems [79]. The human-in-the-loop part was also essential in managing cases in which the automated detection was still in doubt. The study showed that human involvement can help address the generalization issues that automated detection systems face.

Clinical decision support systems represent another important application area where human-in-the-loop design principles inform system architecture. Steffny et al. developed a human-in-the-loop centered AI-based clinical decision support system for professional care planning, emphasizing the importance of designing AI assistance that aligns with clinical workflows and decision-making processes [80]. Theilmann et al. examined success factors for AI in healthcare, identifying human-in-the-loop integration as a key determinant of whether AI systems achieve their intended benefits in clinical practice [89]. These studies highlight that technical performance alone does not guarantee clinical value and that effective integration with human practitioners requires attention to workflow, interface design, and organizational factors.

Notably, the acceptance of human-in-the-loop (HITL) systems by medical professionals can differ substantially from one medical domain to another, as can the type of task. This difference is often more related to the workflow compatibility than the actual model accuracy. In many cases, system deployment failure is related to the presentation of AI results at inappropriate points within the care pathway, increasing documentation burdens, or the uncompensated verification demands placed on the clinician under time pressures. This can result in the system, under these circumstances, reverting to a state of ’rubber-stamping’ or bypassing the system, which can lead to a failure of safety and acceptance. Conversely, the acceptance of the system can be facilitated by the integration with existing decision points, the provision of explanations at a clinically relevant level of granularity, and the establishment of clear escalation and accountability processes [80,89,90]. This evidence suggests that workflow integration is not a secondary implementation detail but a primary determinant of whether healthcare HITL AI delivers real-world benefit.

Healthcare applications in resource-constrained settings present particular challenges and opportunities for HITL AI. Kabata and Thaldar examined human-in-the-loop requirements for AI healthcare applications in low-resource settings, where the scarcity of medical expertise makes AI assistance potentially more valuable but also raises concerns about appropriate oversight [3]. This suggests that Human-in-the-Loop (HITL) design for low-resource contexts should take into consideration the lack of access to expert human evaluators and possibly involve alternative forms of oversight. Fahad and Huang suggested a framework for continuous validation of generative AI in healthcare. The need for this arose from the limitation of sustaining vigilant human oversight in the face of generally reliable AI outputs [32].

The organizational dimensions of human-in-the-loop healthcare AI extend beyond individual clinical encounters to encompass institutional governance and quality assurance. Herrmann and Pfeiffer argued for keeping the organization in the loop as a general concept for human-centered AI, using medical imaging as an illustrative example [90]. Their framework recognizes that effective human oversight requires not only capable individual practitioners but also organizational structures that support monitoring, feedback, and continuous improvement. Griffen and Owens proposed moving from traditional human-in-the-loop models to participatory systems of governance for AI in healthcare, envisioning patient and community involvement in shaping how AI systems are developed and deployed [64].

Emerging applications in pathology and laboratory medicine demonstrate the expanding scope of HITL healthcare AI. Guo et al. evaluated cell AI foundation models in kidney pathology using human-in-the-loop enrichment, developing methods for pathologists to guide model improvement through targeted feedback on challenging cases [91]. Lin et al. applied human-in-the-loop AI screening for hepatic porphyria diagnosis, demonstrating potential improvements over standard diagnostic approaches [92]. Kandala et al. developed cross-lingual mental health ontologies for Indian languages using explainable AI and human-in-the-loop validation, addressing the challenge of extending AI capabilities to underserved linguistic communities [93]. These applications illustrate continuing expansion of HITL approaches into new clinical domains.

### 5.2. Autonomous Systems and Robotics

One such domain where the balance between the capability of artificial intelligence and human oversight is a safety concern is that of autonomous vehicles. In such a case, human-in-the-loop design should be considered on a spectrum of SAE levels of automation, as opposed to a binary concept. Lower levels of automation are typically those where humans are in a constant loop of control assistance, while higher levels of automation leave humans in a role of monitoring with occasional fallback intervention. As levels of automation increase, the bottleneck in human performance shifts from vehicle control proficiency to attention, situational awareness, and preparedness for intervention under time pressure [39].

A key conceptual distinction is between training-time and deployment-time HITL paradigms. Training-time approaches, such as human-guided reinforcement learning and mentor-style correction, use human input to shape policy learning before deployment and to reduce unsafe exploration during development [28,29]. Paradigms related to deployment time, on the other hand, relate to supervisory control, takeover, and response in real-time operational traffic. These paradigms are not interchangeable since they differ in terms of required cognitive resources, failure modes, and levels of regulatory interest. The equivalency of these paradigms obscures critical trade-offs inherent in safety assurance.

Recent empirical work on occupant intervention behavior under extreme driving conditions further underscores this distinction. Xu et al. show that intervention decisions depend on perceived risk trajectory, cue timing, and human confidence in automation status, not only on objective hazard intensity [94]. Related driver-in-the-loop evidence indicates that collaboration quality changes over time as users adapt, which affects both trust calibration and takeover performance [95]. For HITL evaluation, this implies that autonomous-driving systems should be assessed with paradigm-specific metrics (e.g., intervention timing, missed versus unnecessary interventions, recovery quality after takeover) rather than only aggregate task success.

Self-driving laboratories represent an emerging application domain that combines autonomous experimentation with human scientific judgment. Hysmith et al. examined the future of self-driving laboratories, exploring the progression from human-in-the-loop interactive AI to gamification approaches that can engage broader communities in guiding automated scientific discovery [96]. These systems largely automate the experimental procedures while still relying on human scientists to generate hypotheses, interpret results, and control the direction of the research. The human-in-the-loop component ensures that automated experiments align with scientific goals and that unexpected results are properly addressed.

Drone and unmanned aerial vehicle applications have motivated substantial HITL research due to their operational complexity and potential for harm. Lopes conducted studies on operator fatigue, trust, and workload demand in human-in-the-loop AI-enabled drone systems, revealing how extended operation affects human oversight quality [57]. The study also shows that fatigue affects not only trust calibration but also performance, which is a clear indication of the need for effective workload management when it comes to sustaining effective human performance during operations. Inoguchi et al. proposed various workflows for roof damage detection using drones in a collaborative framework between humans and AI [97].

Robotics applications in care and service contexts raise distinctive questions about the appropriate relationship between humans and AI systems. Liu examined human-in-the-loop ethical AI for care robots, drawing on Confucian virtue ethics to develop frameworks for robots that support human flourishing rather than merely performing assigned tasks [75]. Ali explored how human-in-the-loop approaches can enhance safety and adaptability in interactive AI robotic systems, emphasizing the importance of mechanisms for humans to guide and correct robot behavior in dynamic environments [98]. These applications require HITL designs that support nuanced human–robot interaction rather than simple supervisory oversight.

The transportation infrastructure applications of human-in-the-loop (HITL) autonomous systems expand upon the single-vehicle setting by considering broader traffic management objectives. Previati et al. created simulation frameworks for roundabout traffic scenarios that incorporate automated vehicles, artificial intelligence, edge computing, and human-in-the-loop components to study the integration of human oversight in complex traffic scenarios that involve multiple autonomous agents [99]. Happer examined human-in-the-loop versus fully autonomous AI systems for crisis-driven defense electronics manufacturing, analyzing trade-offs between automation efficiency and human adaptability in high-pressure production environments [100].

### 5.3. Cybersecurity and Critical Infrastructure

Cybersecurity is a domain wherein the adversarial nature of cyberthreats and the dynamic nature of cyberattacks present unique challenges for the functioning of AI systems without human intervention. Karunamurthy et al. examined human-in-the-loop intelligence for advancing AI-centric cybersecurity by arguing that cybersecurity requires the integration of AI-based pattern recognition with human expertise on attackers’ motivations and organizational context [81]. Their analysis emphasizes that cybersecurity threats often involve social engineering and exploitation of human factors that AI systems struggle to model, making human judgment essential for comprehensive threat assessment.

The integration of human expertise into AI-driven cybersecurity operations requires careful attention to workflow design and decision support. Owen et al. developed approaches for proactive AI in cybersecurity with human-in-the-loop collaboration for intelligent threat detection and alerting [18]. Their approach utilizes artificial intelligence to prioritize potential threats and offer relevant context to human analysts. The human has the authority to decide the response actions. Turner et al. studied human-in-the-loop decision-making for AI-based cyber defense. The authors examined the interaction between security analysts and AI-based recommendations for cyberdefense. The authors also examined factors that impact human judgment on AI-based cyberdefense [83].

Critical infrastructure protection presents high-stakes applications where the consequences of both successful attacks and false alarms can be severe. Campbell et al. developed human-in-the-loop adaptive AI cybersecurity frameworks for safety-critical infrastructure systems, addressing the challenge of maintaining security while avoiding disruptions caused by overly aggressive automated responses [82]. da Silva examined AI-driven cybersecurity with a human-in-the-loop approach, proposing methods for integrating human expertise into automated security operations centers [101]. These applications require HITL designs that support rapid human response while avoiding alert fatigue that could cause analysts to miss genuine threats.

Software development security has emerged as an application area where generative AI capabilities create both opportunities and risks. Sharma et al. developed cybersecurity-aware human-in-the-loop test orchestration for AI-powered DevSecOps, examining how human oversight can be integrated into automated development pipelines to catch security vulnerabilities before deployment [102]. Konakanchi examined human-in-the-loop secure code synthesis, addressing the challenge of ensuring that AI-generated code does not introduce security vulnerabilities [103]. These applications recognize that AI code generation capabilities must be paired with appropriate security review to avoid introducing new attack surfaces.

### 5.4. Finance, Education, and Industry

This subsection considers the logics of finance, education, and industry, which utilize the same HITL labels, though with different optimization objectives, error cost, and accountability structures. The logics are as follows: finance focuses on legal defensibility and rights protection, education focuses on pedagogical validity and equity, while industry focuses on throughput, quality consistency, and safety. The analytical point is that the effectiveness of the “human-in-the-loop” is not necessarily a guarantee, as its effectiveness depends on the alignment of the points of human intervention with the dominant risks.

With regard to finance, especially in lending and associated decision processes, the primary concern is one of algorithmic accountability rather than the sheer number of human checks. Human override layers can contribute to greater fairness only in combination with decision rationales, explainability for adverse actions, and monitoring for disparate impact across protected groups. Without these components, human checks can be nothing more than a buffer for liabilities while patterns of biased decision processes persist. The governance-focused frameworks developed by Joshi and Singh can only be taken seriously as institutional controls such as documentation governance, escalation governance, and review governance [48,84]. This aligns with broader regulatory debates on meaningful human oversight and accountability design under high-risk AI governance, where policy compliance and substantive fairness can diverge [65,71,72].

The main challenge in the academic setting is how to maintain the legitimacy of assessment while utilizing the potential of artificial intelligence for scalability. Human involvement in the process can help in better judgment; however, this can also lead to instructor inconsistency or institutional bias if the rubrics are not defined well. There is evidence that in all use cases involving grading and content generation, human-in-the-loop design should be aligned to rubrics and moderation for equitable outcomes for different student groups [9,85,86,87,104]. In this setting, human oversight is effective when it is structured as calibrated academic judgment rather than discretionary exception handling.

In industrial or manufacturing settings, human-in-the-loop (HITL) systems are typically assessed for their reliability under production stress. The review process improves defect detection and adaptability to changing environments, but performance suffers when interfaces are overly burdensome cognitively or when operators are relegated to passive monitoring for exceptions. Empirical work on manufacturing and visual inspection settings suggests that successful implementation relies on strong interplay between explainability, training, and feedback mechanisms for continually refining predictive models and practices [23,41]. Across all three domains, the common lesson is that analytical review should focus on how oversight is operationalized—authority, timing, evidence, and feedback—rather than on whether a human is nominally present in the loop.

### 5.5. Cross-Scenario Common Challenges in High-Risk HITL Deployments

In all these domains—healthcare, autonomous systems, cybersecurity, finance, education, and industry—these recurrent problems follow a common pattern despite the varying objectives of each domain. Firstly, there is a problem in establishing accountability in a distributed manner among different models, operators, and organizations. There is a need for clear override privileges and decision logs to avoid diffusion of responsibility. Secondly, trust calibration is another problem that is easily disrupted by over-trust or under-trust. Over-trust leads to automation bias, whereas under-trust leads to a decrease in the effectiveness or usage of the systems. Thirdly, cognitive load and fatigue limit the actual effectiveness of operators in providing oversight.

Fourthly, the timing of human interventions is just as important as the interventions themselves. In other words, delayed or untimely interventions may be just as detrimental to safety despite the formal human-in-the-loop structures that are in place. Fifthly, feedback has a different quality that may include noise, bias, or even strategic behavior that may remain unresolved. This has a significant impact on the reliability of the models. Lastly, there are institutional constraints that affect the feasibility of different types of oversight. Overall, these cross-scenario challenges point to the fact that human-in-the-loop systems need to be viewed as part of an organizational control system rather than just a model–human interface.

## 6. Human-Centered Design and Evaluation

This study examines the viability of human-in-the-loop (HITL) artificial intelligence systems based not only on the capabilities of the artificial intelligence system but also on the level of support provided for human participation in collaborative processes. This section is based on the principles of human-centered design, which are used in the development of interfaces, interactions, and processes that are effective for human participants in collaboration processes. This section outlines principles for designing human-in-the-loop architectures, strategies for monitoring and adapting to humans, and methods for measuring the performance of human-in-the-loop collaboration processes.

### 6.1. Interaction Design Principles

The design of human–AI interaction includes considerations related to the presentation of information, the elicitation of human input, and the design and communication of the allocation of tasks between humans and artificial intelligence. Okuboye examined the redesign of global business processes to optimize the collaboration between artificial intelligence and employees, revealing principles for structuring tasks to take advantage of the strengths of both human and artificial intelligence [10]. The research found that successful HITL implementations require explicit attention to task allocation, with clear delineation of which decisions remain with humans and which are delegated to automated systems. Ambiguity in role boundaries was identified as a significant source of implementation failure.

Prompt design has emerged as a critical interaction design challenge for systems based on large language models. Ranade et al. demonstrated how rhetorical strategies can be systematically applied to design prompts that make AI more useful for human users [31]. Their method involves a human–AI communication concept that can take advantage of the principles of good rhetoric, which are already established. This can change the way prompt engineering is done from a trial-and-error approach to a more communicative approach, which can have implications for how users are taught to use the system.

Attention management represents a fundamental concern in HITL design, particularly for safety-critical applications where lapses in human attention can have severe consequences. Nicosia and Kristensson developed design principles for AI-assisted attention-aware systems in human-in-the-loop safety-critical applications [105]. Their approach tackles the problem of maintaining adequate human vigilance as the AI system performs well in routine cases. This is because it recognizes that the success of the AI system may contribute to the erosion of the human vigilance it originally required. The principles emphasize the design of sustained human engagement rather than simply providing information.

Additional complementary empirical evidence from studies in autonomous vehicles can further clarify these requirements for interaction design. In a study by Xu et al., the authors simulated the riders’ interventions during high-level autonomous driving in extreme conditions and found that the probability of a rider’s intervention depends not only on the actual severity of the hazard but also on the trajectory of perceived risks and the timing of system cues and the occupants’ confidence in the state of the automation [94]. This line of inquiry is socially relevant given observed differences in occupant intervention behaviors, which suggest that some riders are systematically disadvantaged by one-size-fits-all takeover interface designs. In the context of human-in-the-loop (HITL) design, this implies that supervisory interfaces should be designed to incorporate early risk communication, graduated levels of intervention, and fallbacks, rather than relying on late-stage binary takeover requests.

The temporal structure of human–AI interaction affects both the quality of human contributions and the efficiency of collaborative workflows. Ou et al. conducted a case study on revealing and explaining human–AI interaction loop failures, identifying patterns of breakdown that occur when interaction timing, feedback cycles, or task sequencing are poorly designed [106]. Their analysis suggests that one of the most troublesome types of failure modes identified by their model are infinite loops where humans and AI systems are constantly deferring to one another without ever resolving the issue. Effective human-in-the-loop (HITL) design needs to address such failure modes by carefully thinking through the interaction flow and providing explicit mechanisms to resolve deadlocks.

Conversational interfaces present distinctive design challenges for HITL systems that rely on natural language interaction. Sun developed approaches for conversational interfaces cooperating with AI and monitoring technology using human-in-the-loop interaction for intelligent behavioral intervention [107]. The study examines the effects of dialogue design on user engagement, trust, and information exchanged between humans and artificial intelligence systems. The authors of this study, namely Tseng et al., developed AI chatbots for team-based diabetes care by applying these principles through an iterative human-in-the-loop process, showing how this is done in the specific case of healthcare [108].

The comparison of different HITL approaches reveals trade-offs that inform design decisions. Yin et al. compared human-learning HITL approaches with machine-learning HITL approaches in co-design contexts, finding that each design philosophy has distinct strengths depending on task characteristics and user expertise [109]. The human learning approach, in which the artificial intelligence plays a part in guiding human learning or instruction, was found to be more effective for tasks that require skill development. On the other hand, machine learning approaches, in which humans provide training data for the artificial intelligence, were more appropriate for tasks that require adapting to user preferences. A good understanding of these factors can help in determining the appropriate human-in-the-loop configuration for a given application.

User experience design for HITL systems must balance efficiency with factors that sustain human engagement and capability over time. Ahi et al. developed a UX-centric human-in-the-loop system for product lifecycle management that achieved substantial improvements in reviewer productivity while maintaining decision quality [110]. Their approach suggests that productivity improvements should not be at the cost of human skill loss or disengagement. Rather, it is argued that interface design should complement human skills rather than substitute for them. The study proves that careful UX design can lead to not only efficiency benefits but also to sustainable human–AI collaboration.

### 6.2. Human State Monitoring and Adaptive Interfaces

The performance of humans in human-in-the-loop systems is dynamic, depending on their cognitive state, which includes factors such as fatigue, workload, attention, and emotional state. The ability of systems that can monitor and adapt to the state of humans allows for effective collaboration under dynamic states of performance. Lopes carried out extensive research on operator fatigue, trust, and workload demands in AI-based drone systems that are integrated into human-in-the-loop systems [57]. The study revealed a high level of performance degradation in fatigue conditions and identified physiological and behavioral measures that can be used by adaptive systems to detect performance degradation in human capabilities. The results have implications for any human-in-the-loop (HITL) scenario involving human prolonged operation.

Risk management in attention-aware HITL systems requires mechanisms for detecting when human attention has degraded and for intervening appropriately. Nicosia and Kristensson extended their earlier design principles to address risk management in human-in-the-loop AI-assisted attention-aware systems [111]. The framework identifies the type of attention-related risk, the methodologies for monitoring each type of attention-related risk, and the strategies for intervention that range from the least intrusive actions to the most decisive actions. The response to attentional slips detected depends on the level of interest involved as well as the existence of alternative mechanisms.

Adaptive interfaces that respond to detected human states offer the potential for HITL systems that maintain effectiveness across varying conditions. Chivapong examined the strength and challenges of hybrid intelligence approaches, including adaptive mechanisms that adjust AI behavior based on observed human performance [5]. The analysis revealed both technical challenges in effectively inferring human states and design challenges in identifying suitable adaptations. Adaptations that are overly aggressive may compromise human agency, while those that are overly subtle may not effectively address performance degradation.

Workload management in HITL systems involves not only monitoring current workload but also designing task allocation strategies that maintain sustainable human engagement. Benedikt et al. conducted a case study of human-in-the-loop AI in government, examining how workload considerations affect the design and implementation of AI-assisted public services [112]. The study revealed that early deployments were found to underestimate human workload demands, leading to bottlenecks in system performance. Sustainable human-in-the-loop (HITL) deployment requires an appraisal of human capabilities and the establishment of mechanisms for managing human workload demands.

The design of systems for low-literacy or resource-constrained settings presents particular challenges for human state monitoring and adaptive interfaces. Adewale examined human-in-the-loop AI for community health workers, co-designing decision support systems for low-literacy settings [113]. The research emphasized that monitoring and adaptation mechanisms must be appropriate to user capabilities and context, avoiding assumptions about technological familiarity or interface conventions that may not hold across diverse user populations. Inclusive HITL design requires attention to the full range of potential users rather than optimization for typical or expert users alone.

### 6.3. Evaluation Methods and Metrics

The evaluation of HITL systems requires a coherent framework that treats performance as a joint property of humans, AI models, interfaces, and organizational context rather than as model accuracy alone. Building on prior domain studies, we organize evaluation around five dimensions: (i) task effectiveness, (ii) human factors, (iii) interaction process quality, (iv) safety/fairness/governance outcomes, and (v) lifecycle robustness [68,114,115].

For task effectiveness, metrics should include domain performance (e.g., error rate, calibration, quality scores) together with productivity terms such as time-to-decision and throughput. For human factors, evaluation should capture workload, trust calibration, acceptance, and skill retention, because high nominal accuracy can coexist with degraded human vigilance or deskilling. For interaction process quality, useful indicators include intervention timing, override frequency, disagreement resolution quality, and rates of unnecessary versus missed interventions, especially in safety-critical settings [94,95]. For safety, fairness, and governance, systems should be assessed on subgroup disparities, auditability, explanation adequacy, and accountability traceability in real workflows [112,116]. For lifecycle robustness, longitudinal monitoring should test drift sensitivity, adaptation effects, and stability of human–AI collaboration after extended use [46].

Methodologically, this framework implies multi-stage evaluation rather than single-shot benchmarking: controlled experiments for mechanism identification, field studies for workflow realism, and longitudinal audits for sustainability. Experimental comparisons remain essential for isolating effects of interface and explanation design [116,117], while domain-specific studies clarify which metric trade-offs are acceptable in practice [80,118]. Importantly, reporting should make trade-offs explicit: gains in speed or throughput should be interpreted jointly with changes in oversight quality, fairness, and human cognitive burden.

A practical reporting template is to report results as a vector of dimension-wise results rather than an aggregate score. This helps to avoid the suppression of failure modes where one dimension is improving while another is getting worse (e.g., improved decision speed but reduced calibration of human trust). The use of a structured evaluation profile helps to compare results across domains while preserving contextual differences and supports governance decisions related to the readiness of a human-in-the-loop (HITL) system for deployment, restricted deployment, or redesign.

Longitudinal and stress-condition evaluation is therefore essential for revealing adaptation effects, calibration drift, and context-sensitive breakdowns that cross-sectional evaluations can miss. Table 7 summarizes the open challenges and future research directions in human-in-the-loop (HITL) AI across technical, cognitive, organizational, and societal dimensions.

## 7. Governance, Regulation, and Policy

The implementation of artificial intelligence systems in situations that impact basic rights, safety, and welfare has sparked regulatory interest on a worldwide scale, with human oversight being highlighted as a primary need within governance structures. The regulatory needs for human-in-the-loop systems are based on an understanding that AI systems have the ability to cause damage and a belief that humans can help mitigate these risks. This section will discuss the regulatory needs for human oversight, organizational approaches for implementing governance structures, and the political and economic consequences of human-in-the-loop requirements for various stakeholders.

### 7.1. Human-in-the-Loop in AI Regulation

The most comprehensive set of regulations on the human oversight of artificial intelligence systems is provided by the European Union’s AI Act, which outlines different sets of responsibilities depending on the level of risk. According to Article 14 of the EU’s AI Act, human oversight is necessary for high-risk artificial intelligence systems; thus, these systems must be designed in a way that facilitates supervision by natural persons during operation. In her study on the accountability issues that are linked to the imperatives of Article 14 on the supervision of high-risk artificial intelligence systems in the public sector, Constantino found that there are considerable ambiguities [72]. The analysis identified tensions between regulatory demands for meaningful human control and practical constraints on human attention, expertise, and decision-making capacity that affect whether mandated oversight actually improves outcomes.

A broader regulatory comparison is necessary to provide context for these European Union developments. In contrast, in the United States, policy instruments such as Executive Order 14110 and the National Institute of Standards and Technology’s AI Risk Management Framework highlight governance processes, risk documentation, and monitoring, but not a single cross-sector legal architecture. These approaches operationalize oversight through risk functions that govern, map, measure, manage, and lifecycle implementation guidance for agencies, which can inform human-in-the-loop (HITL) development even if language related to “human-in-the-loop” is not as specific as that of Article 14 [119,120]. Stringent requirements are being clearly defined in sector-specific regulatory regimes. In the healthcare sector, for example, the U.S. Food and Drug Administration (FDA) provides detailed recommendations for software as a medical device with respect to AI/ML technologies, which stress the importance of comprehensive controls throughout the entire lifecycle, post-market surveillance, and change management plans. In the context of autonomous vehicles, recommendations from the U.S. Department of Transportation (DOT), National Highway Traffic Safety Administration (NHTSA), stress aspects like development of safety cases, human factors, fallback, and transparent safety assessments [121,122]. Together, these frameworks suggest that regulatory robustness for HITL systems depends on cross-framework alignment between legal accountability, risk-management process maturity, and domain-specific safety governance.

The implementation of regulatory oversight requirements in specific sectors reveals the complexity of translating general principles into operational practice. Lundberg et al. examined human-in-the-loop AI requirements for future unified air traffic management systems, analyzing how aviation safety regulations shape the design of AI-assisted air traffic control [123]. The study proves that the present safety culture and regulatory regime in the aviation industry provide avenues for integrating artificial intelligence while ensuring human accountability. Nevertheless, the development of new artificial intelligence capabilities requires a modification of traditional regulatory practices. Carvell et al. propose a human-in-the-loop testing framework for artificial intelligence agents used in air traffic control systems by using regulated testing methods that explain the means for testing if the artificial intelligence systems meet the regulations for human oversight [124].

Regulatory frameworks must address not only the presence of human oversight but also its effectiveness in achieving intended safety and accountability goals. Fahad and Huang developed a framework for the continuous validation of the results of generative AI systems, which are used for healthcare purposes [32].

The extension of regulatory attention to generative AI systems has created new challenges for human oversight frameworks developed with traditional AI applications in mind. Singh proposed a governance framework for generative AI in banking that operationalizes trust through structured human-in-the-loop oversight at multiple stages of content generation and deployment [48]. The framework focuses on the unique issues that are present when controlling systems that are not fully predictable in their outcomes and whose risk is heavily dependent on the context of use. Anthuvan et al. proposed the Scholarly HI-AI Loop Framework for ethical AI-based knowledge production, which deals with governance issues that are unique to a scholarly environment where issues of integrity and attribution are of primary concern [8].

### 7.2. Organizational Governance

For human oversight to be successful, both competent practitioners and an organizational system that supports monitoring, accountability, and improvement are necessary. The strategy of engaging organizations rather than focusing only on individuals acknowledges that successful oversight is based on organizational capacity and culture, not just the vigilance of individual operators. Chivapong examined the strengths and weaknesses of hybrid intelligence approaches from an organizational perspective, including the factors that affect an organization’s ability to support successful human oversight as artificial intelligence improves [5].

The design of governance frameworks for specific organizational contexts requires attention to sector-specific requirements, existing processes, and stakeholder expectations. Joshi and Vaidya developed a framework for empowering responsible AI adoption in small and medium enterprises, recognizing that resource constraints shape what governance mechanisms are feasible for smaller organizations [76]. The focus is on proportionate governance that provides oversight without creating any burden that may impede the adoption of beneficial AI systems. The concept of AI-DAPT was proposed by Koussouris et al. for data and AIOps integration for future AI development, incorporating human-in-the-loop integration for governance considerations for AI operations in technology environments [125].

Governance frameworks must anticipate and address potential failures in human oversight mechanisms. Sarrat and Gomez examined human-in-the-loop vulnerabilities arising from social engineering at the intersection of AI and critical infrastructure [126]. The analysis showed that the human components in human-in-the-loop systems could be vulnerable to adversarial manipulation, where attackers exploit trust relationships and cognitive biases to compromise the effectiveness of the human oversight. The analysis of the vulnerability shows that there is a need for security considerations for the human oversight mechanisms, in addition to those for the AI systems.

The scalability of human oversight presents governance challenges as AI systems are deployed across larger numbers of decisions and contexts. Ustalov examined challenges in data production for AI with human-in-the-loop involvement, identifying bottlenecks that emerge when human annotation or validation cannot keep pace with AI system requirements [127]. Huang et al. proposed efficient human-in-the-loop active learning as a framework for data labeling that addresses scalability through strategic allocation of human effort [21]. These technical approaches to scalability have governance implications because they affect the feasibility of maintaining meaningful human oversight as AI deployment expands.

### 7.3. Future Governance Directions

The evolution of AI capabilities and deployment contexts continues to challenge existing governance frameworks and to motivate development of new approaches. Hysmith et al. examined the future of self-driving laboratories, exploring governance implications of the progression from human-in-the-loop interactive AI to gamification approaches that engage broader communities [96]. Their analysis suggests that future governance may need to accommodate diverse forms of human involvement beyond traditional expert oversight, potentially including citizen participation in guiding AI development and deployment decisions.

Emerging application domains present governance questions that existing frameworks may not adequately address. Mavrakis et al. examined integration of human-in-the-loop AI to tackle space communication delay challenges, where the physical constraints of space operations require governance approaches adapted to extreme latency and limited communication bandwidth [128]. Rodrigues et al. proposed digital health-enabled community-centered care as a scalable model utilizing human-in-the-loop AI for community health workers, raising governance questions about oversight in resource-constrained healthcare settings [129]. de Miranda explored AI companions for philosophical health using a human-in-the-loop framework, addressing governance considerations for AI applications in personal wellbeing and meaning-making contexts [130].

The development of unified frameworks for HITL governance represents an ongoing research and policy challenge. Tang proposed the Chiron Imperative as a framework of six human-in-the-loop-based systems for creating wise and just AI–human centaurs, offering conceptual resources for thinking about governance across diverse application contexts [15]. Kovalerchuk conducted a critical review of interpretable AI for high-stakes tasks with human-in-the-loop, identifying future trends that will shape governance requirements [4]. Tsiakas and Murray-Rust used human-in-the-loop and explainable AI to envisage new future work practices, examining how governance frameworks must evolve to address changing relationships between humans and AI in workplace contexts [20].

The political economy of the human-in-the-loop requirement can have a bearing on the development of governance. Regulatory requirements to implement a human-in-the-loop mandate may incur a cost of compliance, which may not be favorable to smaller players or those operating in a resource-scarce environment. On the contrary, it may provide a protective cloak to larger players who can afford the cost of governance. Atreyapurapu proposed a human-in-the-loop artificial intelligence framework for scalable online brand protection [131]. Marculescu and Silva examined emerging edge AI for human-in-the-loop cyber-physical systems, highlighting how distributed computing architectures create new governance challenges that centralized frameworks may not address [132]. Atkinson proposed nested human-in-the-loop AI using chain of code prompting for research tool development, illustrating how governance frameworks must accommodate increasingly sophisticated patterns of human–AI interaction [33].

Campbell et al. developed human-in-the-loop adaptive AI cybersecurity frameworks for safety-critical infrastructure systems, addressing governance requirements that arise when AI systems protect essential services [82]. Oye et al. proposed frameworks for mitigating diagnostic errors in AI-driven radiology through human-in-the-loop approaches, demonstrating how governance frameworks must address error management and quality assurance [133]. Assadi and Safaei examined interpretable AI in human–machine systems through the lens of product recommendation engines, revealing governance considerations that arise when AI systems influence consumer decisions at scale [53]. These diverse applications illustrate the breadth of contexts where HITL governance frameworks must operate and the challenge of developing approaches that can accommodate such diversity while maintaining meaningful oversight standards.

## 8. Open Challenges and Future Directions

The previous sections have discussed the current status of human-in-the-loop AI systems with regard to their theoretical underpinnings, technical methodologies, applications, and governance frameworks. The following sections will discuss the challenges that affect the efficacy of HITL systems and also present the possible research directions to mitigate the challenges associated with the technology. The challenges are multifaceted, including technical, cognitive, organizational, and societal aspects, thus reflecting the interdisciplinary nature of human–AI collaboration.

### 8.1. Layered Future Research Agenda Aligned with the HITL Taxonomy

To ensure a more systematic approach for future work, four interrelated layers can be followed and aligned with the aforementioned taxonomy’s dimensions: loop placement and interaction granularity. The technical layer’s priority areas include uncertainty estimation, learning in the presence of disagreement, secure feedback mechanisms, and adaptive escalation strategies. The aforementioned aspects directly affect the loop placement and interaction granularity. The cognitive layer’s priority areas include trust calibration, human workload-aware interface design, and mitigation of human bias and deskilling. The aforementioned aspects affect the interaction granularity. The organizational layer’s priority areas include governance capabilities, staffing models, accountability and traceability, audit processes, and escalation processes. The aforementioned aspects affect the human-in-the-loop design’s viability. The ethical and institutional layer’s priority areas include fairness in heterogeneous feedback mechanisms, transparency requirements, value pluralism management, and sector-specific regulations. The aforementioned aspects affect the human-in-the-loop design’s viability. The aforementioned aspects suggest that future human-in-the-loop research should focus on evaluating the performance of human-in-the-loop methods in terms of not only their predictive performance but also their viability regarding human control calibration at different risk levels and interaction densities.

### 8.2. Scalability of Human Oversight

The scalability of human oversight is one of the key issues in the effective implementation of human-in-the-loop (HITL) technology. The more the AI technology is extended to different decision scenarios and contexts, the more the human oversight capability is limited. The current solutions for the scalability of human oversight in AI technology include active learning techniques for decision scenarios, tiered human oversight for decision scenarios, and sampling-based audit techniques. Future research on human oversight in AI technology should include the development of more sophisticated techniques for identifying scenarios that require human attention and differentiating them from scenarios that can be processed by machines. The development of such techniques can help AI technology in accurately assessing its own uncertainty and can help in the effective allocation of human resources for decision scenarios. The development of different human–AI team configurations can help in identifying different human–AI team configurations that can help in the effective allocation of human resources for decision scenarios. The development of AI technology that can explain itself and identify potential issues can help human overseers in effectively allocating resources for decision scenarios.

### 8.3. Human Factors and Cognitive Limitations

The cognitive limitations of humans are fundamental constraints on the performance of human-in-the-loop systems that cannot be alleviated by technology alone. Fatigue, lapses of attention, cognitive biases, and the bounded nature of human rationality are all factors that can affect the performance of humans in an oversight role. These factors may be exacerbated by the characteristics of the HITL tasks, such as the need to maintain vigilance, repetitive tasks, and the difficulty of maintaining engagement when the AI system is working well. Research on the human factors associated with HITL systems has identified the issues associated with the performance of humans in an oversight role. However, the solution to the problem remains an open issue.

The areas to be addressed in future research on HITL systems are the design of the system to accommodate the limitations of humans and the development of support tools to enhance the performance of humans. Adaptive systems that can respond to the cognitive state of humans by making adjustments to the system to accommodate the limitations of humans are a promising area of research. However, the question of what remedial action to take when a problem is detected is still an open one. Training methods to enhance the skills of humans in an HITL system are an important area of research. This is especially true in the face of the problem of the degradation of skills when AI systems are used. The design of work schedules to maintain the performance of humans over long periods of time is an important area of research.

### 8.4. Conflicting Human Feedback

In human-in-the-loop (HITL) systems that combine the feedback of various contributors, there is a problem of disagreement among the contributors. There is a chance for annotators to assign different labels to similar situations, for stakeholders to hold incompatible views on fairness criteria, and for experts to draw different conclusions on the best course of action. The existing solutions to the problem of dealing with the disagreements of human contributors include majority voting, quality-weighted aggregation of the inputs from the human contributors, and approaches that preserve the information on the disagreements rather than forcing consensus. However, these solutions do not completely address the problem of how to create ground truth in the face of human conflict. The area of conflicting human feedback should be further researched with a differentiation between the cases of human conflict due to ambiguity or plurality of values on the one hand, and human conflict due to errors or lack of information on the other. The development of appropriate tools for detecting the causes of the conflict may help to address the different types of conflict appropriately. Research on the deliberative procedures that help to achieve consensus among humans or that help to understand the nature of the conflict may complement the aggregation-based approaches to human feedback that treat human opinions as given. The impact of conflicting human feedback on the learning of the system and the validity of the AI actions should continue to be an important research question.

### 8.5. Adversarial Manipulation and Security

Human-in-the-loop (HITL) systems incorporate human components that may be vulnerable to adversarial attacks, such as social engineering attacks on trust, cognition, or organizational factors. Attackers may aim to compromise training data by attacking human annotators, evade detection by exploiting human fatigue, or manipulate system behavior by targeting individuals who are involved in feedback provision or deployment decision-making. Hence, the security of HITL systems will not only depend on system security, but also on the robustness of human components against adversarial attacks.

In order to advance HITL security, it is recommended that in the future, threat models for HITL systems are developed, highlighting vulnerabilities in HITL systems resulting from human involvement, as well as proposing countermeasures for different risk contexts. Detection of potential attacks on human components, such as detection of anomalous behavior or feedback, may aid in early detection of attacks on human components in HITL systems. In addition, organizational factors that reduce susceptibility to social engineering attacks should be given more importance in HITL system security, in addition to technical security aspects. Developing HITL systems with security guarantees even when some human components are compromised is a promising yet challenging direction for future research on HITL system security.

### 8.6. Toward Adaptive and Self-Regulating Architectures

The existing state of affairs of human-in-the-loop systems is based on a set of fixed configurations that identify when human input is required during specific junctures of AI system execution. A system that is adaptive in nature, where human input is modulated based on risk levels, AI confidence, or performance, could be seen as offering a better balance between levels of oversight and system efficiency. However, a self-regulating system also poses a unique problem: if left to its own devices, where it is responsible for determining when levels of oversight are required, it is possible that problems of self-assessment could result in levels of oversight being minimized or eliminated altogether. This creates a vicious loop that undermines the normative purpose of a HITL system unless bounded by external limits that are not represented within the model that is being overseen.

A solution that is robust in nature would be to ensure that adaptive triggering is supplemented by non-adaptive measures of oversight. These could be represented by measures that are based on hard levels of oversight for high levels of risk, policy-based intervention rules that are developed by external agencies or organizations, randomized mandatory audit rules, sentinel models that are independent of the AI system, or default levels of oversight that are conservative in nature when uncertainty or changes in probability are represented by levels that are unstable or uncertain. In this regard, risk levels determined by AI represent a means of identifying where levels of oversight are required but do not represent a means of eliminating levels of oversight altogether. Future research should focus on ensuring that meta-oversight is incorporated, where humans govern the levels of adaptation in AI systems, including trigger levels, override levels, and post-incident levels of assessment.

## 9. Conclusions

This survey provides a systematic overview of the field of Human-in-the-Loop AI by discussing its underlying theory, technology, ethics, and practice. The integration of human judgment into the decision-making process in AI systems helps reduce the problems that occur due to the limitations of fully automated systems. At the same time, it presents new challenges for human–AI interaction, management of cognitive load, and designing efficient collaborative systems. A taxonomy for HITL systems is proposed that organizes them based on the position of the loop, granularity of interaction, and temporal characteristics. This helps in comparing different systems and identifying relevant design issues depending on the application domain.

**Figure 5:**
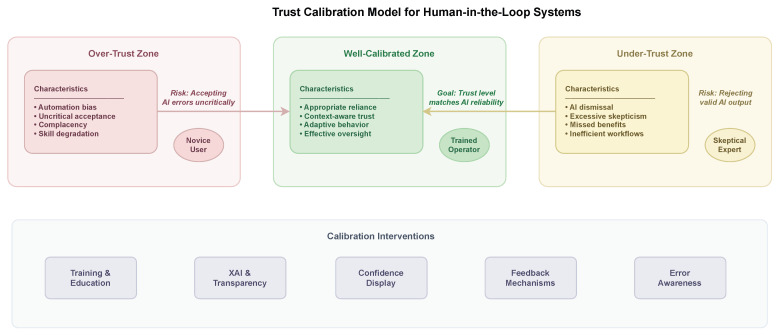
Trust calibration model for Human-in-the-Loop (HITL) systems depicting over-trust, well-calibrated trust, and under-trust states, their associated risks (e.g., automation bias or rejection of valid outputs), and calibration interventions—such as training, explainability, confidence displays, feedback mechanisms, and error awareness—that align human trust with AI reliability. Arrows indicate the direction of trust-state transitions and the influence of calibration interventions on outcomes.

The underlying technology for HITL AI systems has become quite mature with active learning, reinforcement learning from human feedback, and human-in-the-loop generative AI being well-developed fields supported by a wealth of research. Explanatory techniques for AI systems help address the problem of enabling humans to effectively interact with these systems. The problem of trust calibration in these systems has also been addressed by the underlying technology. The application of these techniques in different domains like healthcare, autonomous systems, and cybersecurity highlights the importance of human involvement in decision-making in AI systems and the need for adapting these systems for different domains. Figure 5 summarizes the trust calibration dynamics discussed above, illustrating how different levels of human trust influence interaction with AI systems and how calibration mechanisms can support effective oversight.

Ethical and governance considerations continue to increasingly occupy a central position in human-in-the-loop research. The issues of fairness, bias mitigation, and value alignment continue to be a challenge that human-in-the-loop research can help alleviate but never completely address. The regulatory environment surrounding AI systems, such as the European AI Act, the various initiatives for AI governance in the United States government, as well as sector-specific guidelines such as the FDA AI/ML SaMD guidelines and autonomous vehicle safety guidelines, all indicate the requirements for human oversight and risk mitigation in AI systems that impact society. The organizational level of human-in-the-loop research is an area that continues to deserve attention as the use of AI systems continues to grow exponentially.

There are various challenges that continue to impede the current effectiveness of human-in-the-loop systems. The challenges also indicate avenues for future research. The scalability of human oversight in AI systems, the management of human cognitive biases, and the management of conflicting human opinions, as well as the security of human-in-the-loop systems from adversarial attacks, are all areas that still deserve attention. The design of adaptive architectures that can modulate human oversight based on the level of human oversight required is an area that could help address the scalability of human oversight.

We envision a future where the technology shifts away from the current dominant paradigm of “human-in-the-loop” to more sophisticated “human-with-the-loop” partnerships. These partnerships will be marked by the dynamic distribution of roles, adaptation between human and artificial intelligence partners, and a governance structure to provide accountability without stifling positive innovation. The fundamental design goal is to adjust the level of control provided to the human partner according to levels of risk and uncertainty while maintaining non-negotiable mechanisms to override the AI system and accountability points for high-impact decision-making. Achieving this vision will require sustained collaboration among the fields of computer science, cognitive science, organizational studies, ethics, and policy research. This is particularly true given the high stakes involved in high-impact AI applications. This interdisciplinary collaboration is necessary to ensure that AI systems are aligned with human values and remain under the control of humans.

## Figures and Tables

**Table 1:** HITL method families: required human input, indicative operational cost, core risks, and common failure modes.

Method Family	Human Input Required	Typical Cost	Key Risks	Common Failure Modes
Active learning	Expert/oracle labels Verification of uncertain samples Occasional sampling-policy guidance	Medium–high	Sampling bias Annotator fatigue Privacy exposure in queried items	Myopic query strategy Query/deployment distribution mismatch Annotation artifacts and overfitting to ambiguous cases
Reinforcement learning from human feedback (RLHF)/preference optimization	Pairwise preferences and rankings Critiques and demonstrations Periodic human policy evaluation	High	Reward hacking/specification gaming Norm/preference drift Inconsistent rater judgments	Optimization to proxy signals Truthfulness/helpfulness degradation from over-optimization Collapse to overly safe but uninformative outputs
Interactive machine learning (IML)/human-guided model steering	Continuous corrections and constraints Concept labeling Interactive debugging	Medium–high	Cognitive overload Confirmation bias Inconsistent operator corrections	Non-stationary guidance Oscillatory updates Local patches that degrade global performance
Human-in-the-loop data curation and labeling pipelines	Labeling and adjudication Guideline and gold-set design Iterative error analysis/refinement	Medium–high	Guideline-encoded bias Low inter-annotator agreement Sensitive-information leakage	Label inconsistency/shortcutting Silent label noise Dataset shift as annotation policy evolves
Disagreement-aware label aggregation and adjudication	Multi-annotator labels Annotator metadata and disagreement rationale Expert adjudication for contested items	Medium	False consensus from majority voting Minority-view suppression Unresolved ambiguity propagation	Overconfident hard labels for ambiguous items Escalation bottlenecks Persistent disagreement loops
Post-hoc human validation/escalation (human-on-the-loop)	Output review and approval/override Exception handling Escalation on low-confidence/high-risk cases	Low–medium	Automation bias/rubber-stamping Throughput bottlenecks under peak load Ambiguous accountability	High-risk misses under time pressure Inconsistent overrides Alert fatigue and threshold miscalibration
Human-guided prompt workflows for generative AI	Prompt drafting and refinement Structured output checking Selective fact-checking	Low–medium	Prompt injection Hallucinations and brittle prompt behavior Confidentiality leakage through prompts	Plausible but incorrect outputs Poor reproducibility Failure under adversarial inputs

**Table 2:** Application domains: typical human oversight points, regulatory/standards pressure, evaluation metrics, and common implementation pitfalls.

Domain	Human Oversight Points	Regulation/Standards Pressure	Common Evaluation Metrics	Common Pitfalls
Healthcare (clinical decision support, imaging, triage)	Data curation/labeling Clinician confirmation or override Escalation and audit trails	High (patient safety, medical software/device regulation, privacy)	Sensitivity/specificity, AUROC, calibration (ECE/Brier), subgroup performance, time-to-decision	Site-level dataset shift Spurious correlates Over-trust and weak workflow integration
Autonomous systems (robots, drones, AVs)	Safety-case design and validation Human takeover/teleoperation Incident review	High (safety certification varies by subsystem/jurisdiction)	Safety violations, disengagements/takeovers, edge-case robustness, latency, scenario coverage	Operator over-reliance Delayed takeover/handover failure Untested corner cases and reward hacking
Cybersecurity (detection, triage, response)	Alert triage Analyst feedback loops Playbook approval and post-incident tuning	Medium–high (compliance and critical-infrastructure requirements)	Precision/recall at low FPR, time-to-detect/respond, analyst workload, false-positive burden	Alert fatigue Adversarial adaptation Feedback loops that overfit to SOC routines
Finance (lending, fraud, risk)	Model governance and audits Human review of borderline decisions Adverse-action explanation checks	High (fair lending, consumer protection, auditability)	AUC/KS, expected loss, calibration, fairness metrics, stability/PSI, manual-review rate	Bias amplification/proxy discrimination Concept drift Explanation mismatch and incentive gaming
Legal/public sector (decision support)	Policy design and human adjudication Appeals and override mechanisms Transparency documentation/reporting	High (due process, transparency, accountability)	Error rates by group, calibration, procedural fairness, appeal outcomes, documentation completeness	Legitimacy/opacity concerns Automation bias from historical outcomes Unclear accountability ownership
Industrial quality/manufacturing inspection	Acceptance-criteria and labeling design Human re-check of uncertain items Root-cause feedback loop	Medium (quality/safety standards vary by product)	Defect detection, false rejects, throughput, inspection cost, drift monitoring	Evolving defect taxonomy Inconsistent labels/inspection shortcuts Sensitivity to material/lighting variation

**Table 3:** Human–AI loop configurations in HITL systems. Each configuration represents a distinct relationship between human operators and AI components, characterized by different levels of authority, interaction frequency, and responsibility distribution. The choice among configurations depends on application stakes, AI reliability, regulatory requirements, and available human resources [17].

Configuration	Human Role	AI Role	Authority	Example Context
In-the-Loop	Direct participation in every decision	Supports human decision-making	Human decides	Medical diagnosis, legal decisions
On-the-Loop	Monitors operation, intervenes when necessary	Operates autonomously under supervision	Shared control	Drone surveillance, automated trading
Over-the-Loop	Defines objectives, constraints, and policies	Executes within predefined bounds	Human strategic	Policy systems, organizational AI
Under-the-Loop	Executes final action based on AI input	Provides guidance and decision support	AI advisory	Clinical decision support, recommendations
Along-the-Loop	Parallel collaboration on related tasks	Parallel collaboration with coordination	Lateral coordination	Co-creation, collaborative design

**Table 4:** Technical approaches for incorporating human input in HITL AI systems. The table summarizes the primary mechanisms through which human knowledge, feedback, and oversight are integrated into machine learning workflows, along with the type of human contribution required and representative studies from the literature.

Approach	Mechanism	Human Input Type	Key References
Active Learning	Strategic selection of informative instances for labeling	Annotations, labels	[19,21]
Uncertainty Sampling	Query instances where model confidence is lowest	Correction, validation	[22,23]
Crowdsourced Annotation	Distributed labeling via online platforms	Labels, quality judgments	[24,25]
Expert Annotation	Domain specialists provide specialized labels	Clinical/technical labels	[26,27]
Human Reward Shaping	Direct reward signals based on behavior evaluation	Evaluative feedback	[28,29]
Preference Learning	Pairwise comparisons between alternatives	Relative preferences	[30]
Demonstration Learning	Training via imitation of expert behavior	Task demonstrations	[28,29]
Prompt Engineering	Rhetorical strategies for effective AI communication	Prompt design, refinement	[31]
Iterative Refinement	Multi-round generation with human feedback	Output evaluation, correction	[32,33]

**Table 5:** Trust calibration states in human–AI interaction, associated risks, and interventions for achieving appropriate calibration.

Trust State	Characteristics	Risks	Interventions
Over-trust	Excessive reliance; uncritical acceptance; reduced vigilance	Automation bias; error propagation; skill degradation	XAI [20]; error exposure; confidence displays [11]
Well-calibrated	Context-aware reliance; appropriate skepticism; adaptive behavior	Optimal state	Continuous calibration; transparent uncertainty [55]
Under-trust	Excessive skepticism; rejection of valid outputs	Inefficiency; missed AI benefits; cognitive overload	Demonstrated reliability; transparency [56]

Note: Moderating factors include fatigue [57], political preferences [58], prior experience, and individual differences.

**Table 6:** Application domains for HITL AI systems with characteristic configurations and challenges. High-risk domains typically require tighter human oversight due to potential consequences of errors, while medium-risk domains may employ more flexible configurations balancing oversight with operational efficiency.

Domain	Risk Level	Typical Loop Config	Key Challenge	Representative Studies
Healthcare	High	In-the-Loop	Clinical accountability; diagnostic validation	[26,78,79,80]
Autonomous Systems	High	On-the-Loop	Real-time safety; human takeover capability	[28,29,39,57]
Cybersecurity	High	Along-the-Loop	Scalability; adversarial adaptation	[18,81,82,83]
Finance	High	Over-the-Loop	Regulatory compliance; fraud detection	[45,48,84]
Education	Medium	In-the-Loop	Fairness; pedagogical quality; assessment validity	[9,85,86,87]
Manufacturing	Medium	On-the-Loop	Efficiency; quality inspection accuracy	[13,23,41]

**Table 7:** Open challenges and future research directions in HITL AI. The table summarizes persistent limitations affecting HITL system effectiveness and outlines research directions that may address these challenges across technical, cognitive, organizational, and societal dimensions.

Challenge	Description	Current Approaches	Future Research Directions
Scalability of Human Oversight	Human capacity insufficient for AI decision volume at scale	Active learning; tiered oversight; sampling-based audits	Uncertainty quantification; AI self-assessment; team configurations
Human Cognitive Limitations	Fatigue, attention lapses, cognitive biases affect oversight quality	Workload management; training programs; interface design	Adaptive systems responding to cognitive state; sustainable work structures
Conflicting Human Feedback	Disagreement among annotators and stakeholders	Majority voting; weighted aggregation; quality metrics	Deliberative approaches; disagreement characterization; consensus methods
Adversarial Manipulation	Social engineering targeting human components	Technical security measures; access controls	HITL-specific threat models; manipulation detection; procedural safeguards
Adaptive Architectures	Fixed configurations may not match varying needs	Predetermined human involvement points	Risk-based dynamic adjustment; self-regulating systems; meta-level oversight

## Data Availability

No new datasets were created or analyzed in this study. Data sharing is not applicable to this article because it is a survey based on published literature.
